# Integrated multi-omics analysis of RB-loss identifies widespread cellular programming and synthetic weaknesses

**DOI:** 10.1038/s42003-021-02495-2

**Published:** 2021-08-17

**Authors:** Swetha Rajasekaran, Jalal Siddiqui, Jessica Rakijas, Brandon Nicolay, Chenyu Lin, Eshan Khan, Rahi Patel, Robert Morris, Emanuel Wyler, Myriam Boukhali, Jayashree Balasubramanyam, R. Ranjith Kumar, Capucine Van Rechem, Christine Vogel, Sailaja V. Elchuri, Markus Landthaler, Benedikt Obermayer, Wilhelm Haas, Nicholas Dyson, Wayne Miles

**Affiliations:** 1 Department of Cancer Biology and Genetics, The Ohio State University, Columbus, OH USA; 2 The Ohio State University Comprehensive Cancer Center, The Ohio State University, Columbus, OH USA; 3 Massachusetts General Hospital Cancer Center, Charlestown, MA USA; 4 Harvard Medical School, Boston, MA USA; 5 Berlin Institute for Medical Systems Biology, Max-Delbrück-Center for Molecular Medicine in the Helmholtz Association, Berlin, Germany; 6 Department of Nanobiotechnology, Vision Research Foundation, Sankara Nethralaya, Chennai, Tamil Nadu India; 7 Department of Pathology, Stanford University, Stanford, CA USA; 8 Center for Genomics and Systems Biology, Department of Biology, New York University, New York, USA; 9 IRI Life Sciences, Institute für Biologie, Humboldt Universität zu Berlin, Berlin, Germany; 10 Core Unit Bioinformatics, Berlin Institute of Health (BIH), Berlin, Germany; 11 Present Address: Agios Pharmaceutical, Cambridge, MA USA

**Keywords:** Tumour-suppressor proteins, Transcriptomics, Cancer metabolism

## Abstract

Inactivation of RB is one of the hallmarks of cancer, however gaps remain in our understanding of how RB-loss changes human cells. Here we show that pRB-depletion results in cellular reprogramming, we quantitatively measured how RB-depletion altered the transcriptional, proteomic and metabolic output of non-tumorigenic RPE1 human cells. These profiles identified widespread changes in metabolic and cell stress response factors previously linked to E2F function. In addition, we find a number of additional pathways that are sensitive to RB-depletion that are not E2F-regulated that may represent compensatory mechanisms to support the growth of RB-depleted cells. To determine whether these molecular changes are also present in *RB1*^*−/−*^ tumors, we compared these results to Retinoblastoma and Small Cell Lung Cancer data, and identified widespread conservation of alterations found in RPE1 cells. To define which of these changes contribute to the growth of cells with de-regulated E2F activity, we assayed how inhibiting or depleting these proteins affected the growth of *RB1*^*−/−*^ cells and of *Drosophila* E2f1-RNAi models in vivo. From this analysis, we identify key metabolic pathways that are essential for the growth of pRB-deleted human cells.

## Abstract (web summary)

Swetha Rajasekaran et al. integrate transcriptional, proteomic, and metabolomic data to explore how cells adapt to inactivation of RB1, a hallmark of cancer. Combined with their genetic analyses in a *Drosophila* model, the authors identify key metabolic pathways that may be involved in the growth of RB1-depleted cancer cells.

## Introduction

pRB, the protein product of the Retinoblastoma susceptibility gene (*RB1*), and E2 promoter binding factor (E2F) have important and well-described roles in cell cycle control. pRB binds to the activator E2Fs (E2F1-3) and one inhibitory E2F, E2F4^1^, and in collaboration with other pRB-family members and E2Fs orchestrates the tight transcriptional control of E2F target genes^2^. Through this activity, pRB functions to obstruct the expression of E2F target genes that control cell cycle progression^3,4^, metabolism^5^, and mitochondrial function^6^. Inactivation of this regulatory process is one of the hallmarks of cancer cells. The various mechanisms that disrupt pRB activity in tumor cells include the loss or silencing of Cyclin-Dependent Kinase (CDK) inhibitors, CDKN2A-D (p15, p16, p18, and p19)^7,8^, amplification or over-expression of CDK4^9^, 6^10^ or Cyclin D1 (CCND1)^11–14^, direct mutation the *RB1* gene^15–17^, or expression of viral oncoproteins that target pRB^18,19^. Irrespective of the mechanism of inactivation, pRB-loss deregulates E2F-dependent transcription.

Despite clear evidence of widespread disruption of the pRB pathway in human cancers, our understanding of the ways that pRB inactivation changes the molecular properties of human cells is limited. The pRB literature has been dominated by transcription studies and these have focused primarily on the changes seen at canonical E2F targets^20^ and at genes encoding known cell cycle regulators. However, a number of recent publications have expanded the biological functions and mechanisms of action of pRB. In particular, these studies have identified novel roles for pRB in mitochondria function^21^, new pRB-interacting partners^22^, and examined the overall changes to the proteome that appear in *Rb1* mutant mouse tissues^23^. These proteome profiling experiments revealed, unexpectedly, that a major impact of *Rb1* ablation on the proteome involves changes in the levels of mitochondrial proteins^23^. However, the full extent of the proteomic changes caused by RB1-deletion was masked by a high degree of sample-to-sample variation between organisms, a weakness that is inherent to the profiling of tissue samples that contain complex mixtures of cell types.

To obtain a picture of how pRB-loss changes human cells, we generated a detailed and quantitative map. For this, we measured how pRB-depletion alter the transcriptome, proteome, and metabolome of cultured diploid non-tumorigenic Retinal Pigment Epithelial 1 (RPE1) cells. We then compared these changes to molecular events in *RB1*^*−/−*^ Retinoblastoma tumors and Small Cell Lung Cancer (SCLC) to understand how alterations within model pRB-depleted RPE1 cells compared to primary patient samples. Many of the changes we identify are directly linked to dysregulated E2F function; however, a number of pathways with limited links to E2F were also found. These findings suggest that pRB-depletion affects both direct and indirect mechanisms that prime cells for neoplastic growth. To interrogate which of these changes were essential for the growth of cells with de-regulated E2F function, we used an established E2f1-RNAi model in *Drosophila*^24^. This data identified a collection of pathways that are altered in pRB-depleted cells and important for the cellular growth of these cells and highlight the complex and integrated cellular reprogramming caused by pRB-depletion in cells and organisms.

## Results

### pRB-deletion results in widespread cellular changes

**Fig. 1:**
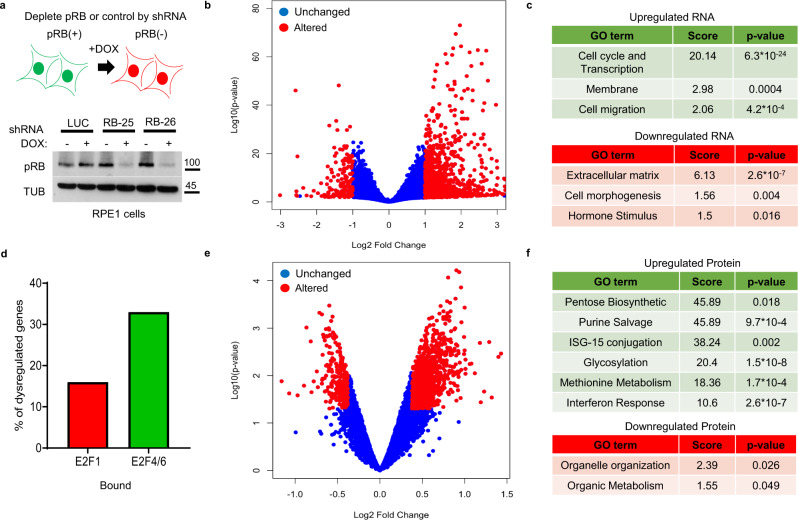
Profiling how pRB-depletion changes human RPE1 cells. **a** Schematic detailing experimental approach and western blot of pRB and Tubulin (TUB) from RPE1 cells infected with DOX-inducible shRNAs targeting Firefly Luciferase (LUC) or pRB (RB-25 and RB-26). **b** Log2 fold-change of RNA changes in RPE1 cells depleted of pRB (RB25 and RB26), Red dots are statistically significant. **c** Gene ontology analysis of upregulated mRNAs (Green box, enrichment scores, and FDR *p*-values) and downregulated mRNAs (Red box, enrichment scores, and FDR *p*-values). **d** Graph of genes changed upon pRB-depletion from RPE1 cells that are bound by E2F1 or E2F4/6 from ChIP data. **e** Log 2 Protein fold-change from RPE1 cells depleted of shRNAs targeting RB using shRNA RB25 and RB26, Red dots are statistically significant. **f** Gene ontology analysis of upregulated proteins (Green box, enrichment scores, and FDR *p*-values) and downregulated proteins (Red box, enrichment scores and FDR *p*-values).

To assess the effects of pRB-depletion, we asked how the knockdown of pRB alters the transcriptome, proteome, and metabolome of RPE1 cells. RPE1 cells were chosen as they are TERT-immortalized (but not transformed) human cells that contain wild type and active pRB (Supplementary Fig. 1A) and p53. RPE1 cells have been used in many previous studies of pRB function and are known to display changes in transcription^4,25^, chromatin organization^26,27^, and cellular metabolism^5^ in response to pRB loss. pINDUCER11 Doxycycline (DOX) inducible short hairpins (shRNAs) were employed to deplete pRB or a Firefly Luciferase (FF) control sequence^28^. RPE1 cells were infected with the respective pINDUCER11 constructs and FACS sorted using GFP to establish pools of stable cells. For these experiments, we used two previously published shRNAs that target different regions of the RB1 mRNA, RB25 and RB26^28^, and a negative control sequence targeting Firefly (FF) Luciferase (LUC). To determine the effects of pRB-depletion on the transcriptome, we first subtracted genes that showed changed levels in control cells that express Firefly Luciferase (FF), upon DOX treatment. We then determined the baseline (−DOX) transcriptome from FF, RB25, and RB26 and found no statistically significant difference. Next, we compared the levels of gene expression in pRB-depleted cells with their non-Doxycycline counterparts. This enabled the removal of mRNA changes caused by the addition of DOX and/or the production of the FF shRNA (LUC), and to identify changes common to both pRB-targeting shRNAs. To deplete pRB or LUC, we treated the respective RPE1 lines with DOX and tracked shRNA expression (RFP) and pRB via western blot. From this analysis, we find very homogenous RFP levels in DOX-treated cells, suggesting even shRNA production (Supplementary Fig. 1B) and reduced pRB levels after 6 days of DOX treatment (Fig. 1a, Supplementary Fig. 1C, D). Strong shRNA-mediated depletion of pRB was found in DOX-treated cells, independent of confluency (Supplementary Fig. 1E). Cells at 90% confluency were selected for this analysis. We next checked the protein levels of the pRB homologs, RB Transcriptional Corepressor Like 1 (RBL1) or 2 (RBL2) following DOX treatment. From this analysis, we found RBL2 levels unchanged and increased levels of the E2F target, RBL1 (Supplementary Fig. 1F). In agreement with previous studies^23^, we do not find altered growth rates (Supplementary Fig. 2A) or cell cycle progression of pRB-depleted cells (Supplementary Fig. 2B–E). We then used these cells to profile both DOX-treated and untreated cells to measure mRNA changes using RNA-sequencing (RNA-seq), the proteome using Tandem Mass Tag Mass Spectrometry (TMT-MS) and the metabolome with Liquid chromatography–mass spectrometry (LCMS).

To determine the effects of pRB-depletion on the transcriptome, we first subtracted genes that showed changed levels in FF cells upon DOX treatment. We then determined the baseline (−DOX) transcriptome from FF, RB25, and RB26 and found no statistically significant difference. Next, we compared the levels of gene expression in pRB-depleted cells with their non-Doxycycline counterparts. This enabled the removal of mRNA changes caused by the addition of DOX and/or the production of a non-specific FF shRNA (LUC), and to identify changes common to both pRB-targeting shRNAs. This analysis identified 990 genes that were upregulated in pRB-depleted cells and 164 that were downregulated (Fig. 1b) (Significant genes are FDR *p*-value <0.05 with log2 fold-change >1) (Supplementary Data 1). FDR-adjusted Gene Ontology (GO) analysis identified gene groups that were most sensitive to pRB-loss. In agreement with previous reports, the expression of cell cycle factors and the transcription machinery were significantly upregulated (6.3 × 10^−24^) upon pRB-depletion (Fig. 1c). Changes were also evident in additional categories, including membrane genes and cell migration (Fig. 1c). Many of these genes are known to be E2F targets genes^29^ (Fig. 1d).

To quantify the effects of pRB-depletion on the proteome of RPE1 cells, the same panel of cells was profiled using multiplexed quantitative proteomics using tandem mass tag (TMT) reagent technology and the SPS-MS3 method for accurate quantification^30–32^. The levels of 6551 proteins were quantified in all samples. In accordance with our RNA-seq approach, the protein levels in pRB DOX-treated cells were compared to untreated and normalized against FF DOX+ vs. DOX-. As illustrated in Fig. 1e, pRB-knockdown caused widespread changes to the proteome (Supplementary Data 2). FDR-adjusted GO analysis of the 527 proteins that were significantly (*p* < 0.05) changed in pRB-depleted cells, identified a subset of pathways that differed from the set highlighted by the RNA analysis. Protein pathways upregulated in the pRB-depleted cells include pentose biosynthesis, purine salvage, glucose and methionine metabolism, and the interferon response (Fig. 1f). Only two GO groups are presented in the proteins downregulated after pRB-knockdown: organelle organization and organic metabolism (Fig. 1f).

### Independent experimental testing of omics data

**Fig. 2:**
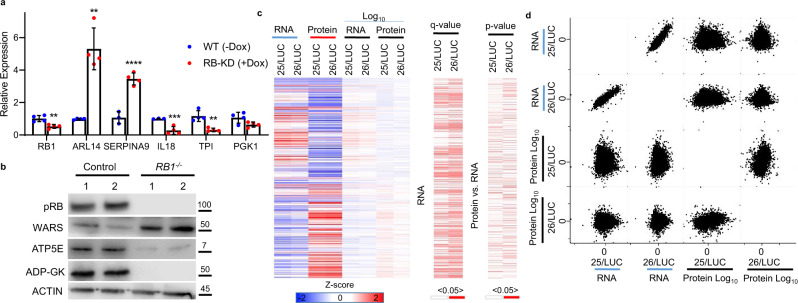
Combining profiles to determine the effect of pRB-depletion. **a** Relative RT-PCR levels of RB1, ARL14, SERPINA9, IL18, TPI, and PGK1 from RB-25/26 without DOX addition (WT) and plus DOX (RB-KD) (**p < 0.01, ***p < 0.001). *n* = 3 biological replicates and a total of 9 technical replicates. Error bars represent standard deviations from the replicates. **b** Western blots of pRB, WARS, ATP5E, ADP-GK and ACTIN from control and *RB1*^*−/−*^ RPE1 cells. **c** Heat maps and statistical analysis of RNA and protein changes in pRB-depleted cells. **d** Correlations comparing RNA and protein changes between replicates, highlighting the poor correlations between RNA and protein.

These profiles highlight that pRB-depletion results in widespread cellular changes. To independently evaluate these changes, we first tested the levels of a subset of genes that show altered expression following pRB-depletion using RT-PCR. For this analysis, we selected two upregulated, ARL14 and SERPINA9, and four downregulated genes, RB1, IL18, TPI and PGK1, from our RNA-seq data for independent testing. RT-PCR of these genes from control and pRB-depleted cells, produced homologous results to the RNA-seq data (Fig. 2a). Alterations in apoptotic genes are common in pRB-depleted cells, and our multi-omics analysis found these genes upregulated at the RNA but not the protein level. To confirm these observations, we conducted RT-PCR for cell death genes and found their RNA increased in pRB-depleted cells (Supplementary Fig. 2F). Western blot analysis of candidate proteins sensitive to pRB-depletion identified by Mass Spectrometry, highlighted the reproducibility of our data (Fig. 2b). To determine how each of the changes in RNA and protein levels are coordinated in pRB-depleted cells, we conducted a system-wide analysis. The levels of RNA and protein were converted in log10 values from individual RNA-seq and proteomic replicates and then compared. This analysis showed that alterations in the levels of RNA, rather than protein, were the most significant between control and pRB-depleted cells (Fig. 2c). We next investigated how well RNA and protein changes are linked in each replicate. From this, we found that transcriptional profiles of RB25 and RB26 were very tightly correlated (compare RNA: RB25 vs. RB26) (Fig. 2d) whilst the protein profiles also showed strong correlations (compare Protein: RB25 vs. RB26) (Fig. 2d). However, merging the RNA and protein datasets from either shRNA group do not correlate well (compare RNA and Protein: RB25 vs. RB26) (Fig. 2d). These results suggest that RNA changes upon RB-loss are frequently not reflected at the protein levels in RPE1 cells. To determine whether these changes were affected by codon bias or rare amino acid composition, we analyzed the relative level of each in the proteins altered by pRB-depletion. From this analysis, we found that codon or amino acid biases are unlikely contribute to these effects (Supplementary Fig. 3A, B). This analysis shows that pRB-depletion causes complex alterations in both RNA and protein levels and synthesis rates.

### Metabolic changes are widespread in pRB-depleted cells

**Fig. 3:**
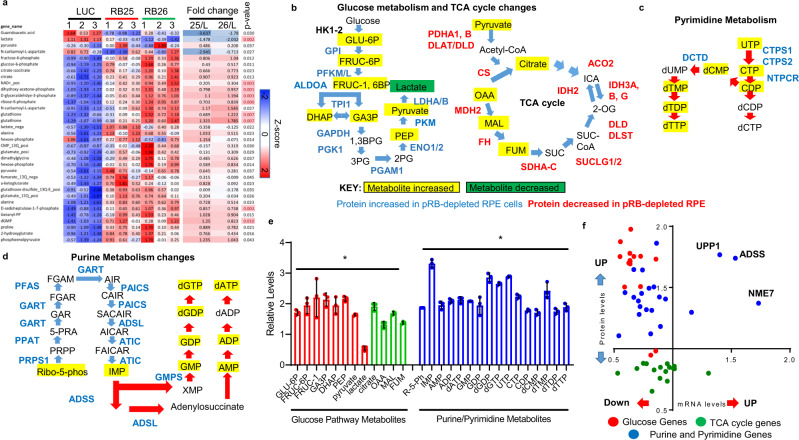
Glucose and purine/pyrimidine metabolism changes in pRB-depleted cells. **a** Heat map of metabolic changes in LUC, RB25, and RB26 DOX-treated/DOX-untreated with fold changes and adjusted *p*-value calculations. **b** Pathway analysis of Glucose metabolism and TCA cycle genes upon pRB-loss. Upregulated metabolites highlighted in yellow, downregulated metabolites shown in green, protein increased in pRB-depleted cells shown in blue and proteins decreased in pRB-depleted RPE1 cells shown in red. **c** Pathway analysis of Pyrimidine metabolism upon pRB-loss. Upregulated metabolites highlighted in yellow, protein increased in pRB-depleted cells shown in blue. **d** Pathway analysis of Purine metabolism upon pRB-loss. Upregulated metabolites highlighted in yellow, protein increased in pRB-depleted cells shown in blue. **e** LCMS data showing relative (pRB (−)/pRB (+)) metabolite changes from RPE1 cells for Glucose pathway metabolites (red bars) and Purine/Pyrimidine metabolites (blue bars) (*p < 0.01). *n* = 4 biological replicates and a total of 8 technical replicates. Error bars represent standard deviations from these replicates. **f** RNA and Protein changes in glucose metabolism genes (red circles), purine and pyrimidine metabolism genes (blue circles), and TCA cycle genes (green circles) in RPE1 cells depleted of pRB.

We have previously described mitochondrial defects in *Rb1*-deleted murine tissue^23^ and asked whether similar changes also occur in human cells. Steady-state LCMS profiling of the panel of RPE1 cells identified 35 metabolites (Fig. 3a) that were altered in pRB-depleted cells (Supplementary Data 3). Many of these changes affected metabolites in the glucose metabolism and purine/pyrimidine biosynthetic pathways. In concordance with previous reports, we observed a clear and consistent alteration in mitochondrial processes in pRB-depleted human cells (Supplementary Fig. 3C, D). To independently evaluate our results, we tested the levels of cellular acidification, using ELISAs from DOX-treated cells. From this analysis, we found elevated levels in pRB-depleted cells (Supplementary Fig. 3E). To measure how pRB-depletion changes the metabolic profile of different cell types, we used pLKO.1 vectors and different sets of shRNAs to deplete pRB from human BJ fibroblasts. As shown in Supplementary Fig. 3F, shRNA-mediated pRB-knockdown resulted in highly reproducible metabolite changes suggesting that these alterations are in direct response to pRB-depletion. These changes were not seen in cells with deletions in pRB homologs, *Rbl1* and *Rbl2* (Supplementary Fig. 4A). To determine whether changes in mTOR activity, caused by pRB-depletion, was contributing to this process, we measured the phosphorylation levels of the mTOR target, S6 kinase. These results showed mTOR levels are unchanged in pRB-depleted cells (Supplementary Fig. 4B). To better understand the associated metabolic consequences, we combined the metabolic, and proteome profiles and looked for concerted patterns of change in the merged data. We discovered that many of the proteins and metabolites involved in the glucose metabolism pathway are coordinately increased in pRB-depleted cells (Fig. 3b, e (Red bars) and F, Red dots). The majority of these genes were unchanged or decreased at the mRNA level (Fig. 3f, Red dots), despite being directly bound by E2F1 (Supplementary Fig. 4C). This suggests that the elevated protein levels may reflect increased metabolite levels (Fig. 3b, e, f). In contrast, although multiple TCA cycle metabolites were increased in pRB-knockdown cells (Fig. 3b, e (Green bars), the proteins that catalyze these reactions displayed reduced levels (Fig. 3b, f (Green dots)). Decreased enzymatic activity, coupled with elevated amounts of metabolites, are consistent with decreased movement through TCA cycle previously described in pRB mutant cells^23^. Together, these results describe an extensive pattern of metabolic reprogramming of the glucose pathway and TCA cycle following pRB-depletion from human cells^33^.

In a similar way, the combination of proteomic and metabolic data revealed a consistent and extensive pattern of changes in purine and pyrimidine metabolism in pRB-depleted cells, with both synthetic pathways clearly elevated by pRB-knockdowns. The profiles showed increased levels of multiple key components of pathways leading to the production of nucleosides and nucleotides (highlighted in blue and yellow in Fig. 3c, d). Concordantly, metabolite profiling of pRB-depleted cells showed increased levels of the purine and pyrimidine precursors detected in our analysis (Fig. 3e, Blue bars), with the exception of dCDP and dCTP. This systematic pattern of changes in enzyme levels was evident even though many of the purine and pyrimidine genes were not transcriptionally upregulated upon pRB-depletion (Fig. 3f, Blue dots). This suggests that the increased protein levels reflect the elevated activity of the synthetic pathways, rather than being solely a consequence of increased transcription. Chromatin Immunoprecipitation (ChIP) of the activator E2F, E2F1, confirmed that several of the purine and pyrimidine genes were bound by E2F1 (Supplementary Fig. 4D) but many of these were not transcriptionally upregulated upon pRB-depletion (Fig. 3f, Blue dots). To exclude the possibility that the activation of purine and pyrimidine metabolism upon pRB-knockdown was a unique feature of RPE1 cells or the pINDUCER11 system, we independently evaluated pRBs role in purine and pyrimidine metabolism. By profiling shRNA depleted human BJ fibroblasts, we identified widespread changes in purine and pyrimidine metabolites (Supplementary Fig. 3F). Taken together, these findings provide strong evidence that the purine/pyrimidine pathway is strongly upregulated in pRB-depleted cells. Such changes are consistent with increased replication and/or DNA damage.

### Metabolic changes identified in pRB-depleted RPE1 cells are also present in *RB1*^*−/−*^ Retinoblastoma

**Fig. 4:**
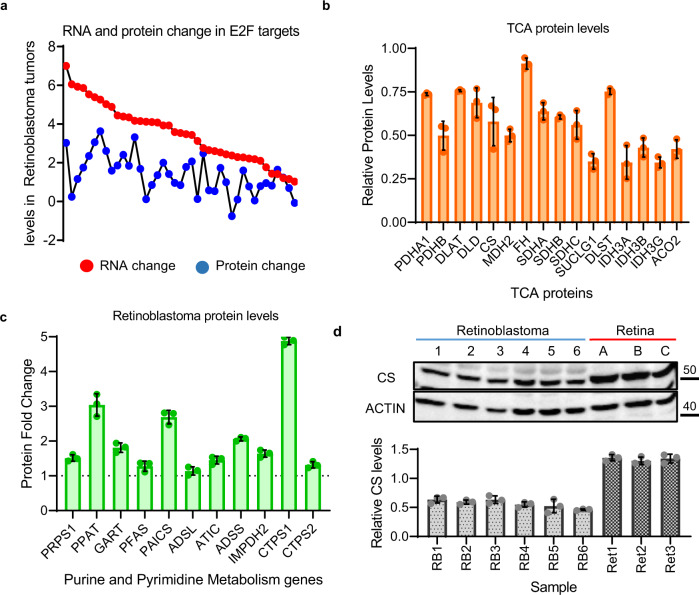
Comparing the effect of pRB-depletion in RPE1 cells to *RB1* mutation in Retinoblastoma tumors. **a** Comparison of the RNA and protein levels of E2F target genes in *RB1*^*−/−*^ Retinoblastoma tumors. **b** Relative TCA cycle protein levels in Retinoblastoma tumor samples compared to normal retina. *n* = 5 independent tumors and 5 retina controls. Error bars represent standard deviations from these replicates. **c** Relative purine and pyrimidine protein levels in Retinoblastoma tumor samples compared to normal retina. *n* = 4 independent tumors and matched controls. Error bars represent standard deviations from these replicates. **d** Western blots of the TCA cell cycle gene, CS, and ACTIN, from six Retinoblastoma tumors (1–6) and three retinal samples (**a**–**c**). Graph represents western blot quantification from three independent assays. Error bars represent standard deviations from these replicates.

While RPE1 cells are a good model for pRB research, we next wanted to investigate whether these changes are also seen in *RB1*-mutant human tumors. To assess this, we compared the RNA and protein changes seen in RPE1 cells with the properties of *RB1*^*−/−*^ human Retinoblastomas. We utilized previously published RNA-seq^34^ and proteomic^35^ data from normal retinal tissue and Retinoblastoma tumors to investigate how the RNA and protein levels of E2F target genes were changed. In agreement with our RPE1 data, we find poor correlations between relative RNA and protein changes in many E2F target genes from these tumors (Fig. 4a). To independently determine the RNA expression changes of E2F target genes in Retinoblastoma, we used RT-PCR to measure the levels of a subset of E2F targets. By analyzing this independent tumor cohort, we find strong upregulation of E2F target genes in these tumors (Supplementary Fig. 4E, F). We then expanded this analysis to ask whether the protein levels of the metabolic pathways were also altered. From this, we found that much like our RPE1 cells, TCA cycle components were lower in *RB1*^*−/−*^ mutant tumor samples (Fig. 4b), whilst the proteins necessary for enhanced purine and pyrimidine biosynthesis were elevated (Fig. 4c). We next independently tested whether we would detect these protein changes using western blot analysis. To do this, we measured the levels of TCA protein, Citrate Synthase (CS), in six Retinoblastoma tumor samples and three normal retinal controls. In agreement with our proteomic data, we find reduced levels of the CS protein in each of the tumor samples (RB1-6) compared to the controls (Ret1-3) (Fig. 4d). These results suggest that our profiles of pRB-depleted RPE1 cells mirror key changes present in *RB1*^*−/−*^ Retinoblastoma tumors.

### Proteomics changes in pRB-depleted cells are conserved in *RB1*^*−/−*^ mouse tissue and human tumors

**Fig. 5:**
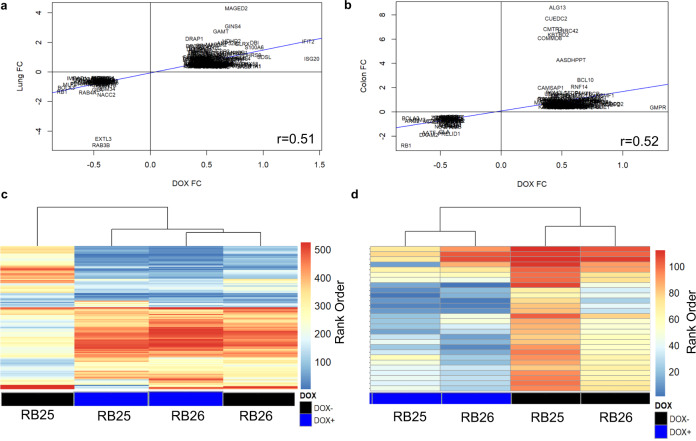
Proteomic changes in RB-depleted RPE1 cells are also present in *Rb1*^−/−^ murine tissue and SCLC tumors. **a** Graph and correlation of protein changes in pRB-depleted RPE1 cells relative to *Rb1*^*−/−*^ murine lung tissue. **b** Graph and correlation of protein changes in pRB-depleted RPE1 cells relative to *Rb1*^*−/−*^ murine colon tissue. **c** Heat map of proteins altered in pRB-depleted RPE1 cells compared to the proteome of SCLC tumors. **d** Heat map of upregulated and statistically significant proteins SCLC tumors compared to pRB-depleted cells.

To determine whether changes in pRB-depleted cells are also seen in complex tissue and adult human tumors, we compared the proteome of pRB-depleted RPE1 cells to those from *Rb1*-mutant tissue and human SCLC tumors. For this, we first examined the correlation between protein changes in pINDUCER pRB-depleted RPE1 cells to *Rb1*-deleted murine tissue^23^. From this, we identified a strong correlation (r = 0.51) between protein alterations in pRB-depleted RPE1 cells relative to both *Rb1*^*−/−*^ lung (Fig. 5a) and colon (Fig. 5b) mouse tissue. The reproducibility of protein changes in experimental models of pRB-depletion, suggests that these models provide important and conserved information on pRB’s role in cellular regulation. We then expanded this analysis to determine how these profiles compared to published proteomic datasets from human *RB1*^*−/−*^; *TP53*^*−/−*^ SCLC tumors^36^. By comparing statistically significant differences from pRB-depleted cells to SCLC tumors, we find a clear separation of proteomic signature between pRB-knockdown (+DOX) relative to pRB-positive (−DOX) cells (Fig. 5c). We then asked whether proteomic changes (Fold-change > 2, *p*-value > 0.05) in SCLC tumors were also present in our RPE1 cells. As shown in Fig. 5d, these changes are also seen in our cells, suggesting that our RPE1 based on pRB-depletion is able to capture proteomic events seen in *RB1*^*−/−*^ tumors. From these results, our data suggest that RPE1 accurately models how pRB-knockdown changes the biology of cells and provides a basis for tumorigenesis.

### Identifying sensitivities to proteomic changes

**Fig. 6:**
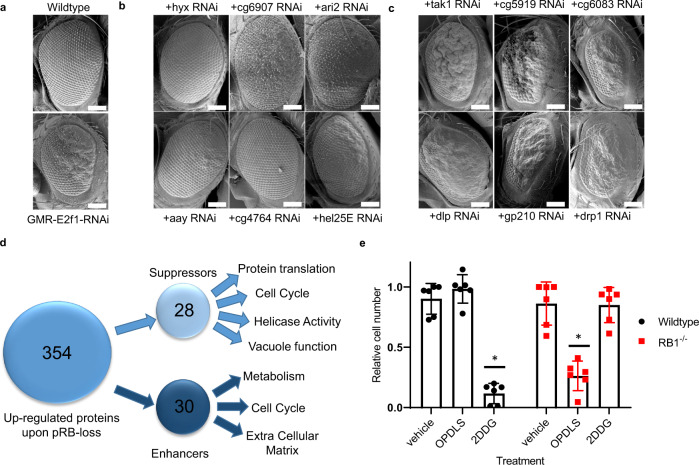
Identifying the essential proteins within the upregulated protein group using in vivo models. **a** Representative Scanning Electron Microscopy (SEM) images of *Drosophila* eyes from Wild type and GMR-E2f1-RNAi animals. White bar = 100 µm. **b** Representative Scanning Electron Microscopy (SEM) images of *Drosophila* eyes from genes that when depleted can suppress the GMR-E2f1-RNAi phenotype. White bar = 100 µm. **c** Representative Scanning Electron Microscopy (SEM) images of *Drosophila* eyes showing seven genes that when depleted can enhance the GMR-E2f1-RNAi phenotype. White bar = 100 µm. **d** Schematic detailing the number of modifiers identified in the GMR-E2f1-RNAi genetic screen. **e** Relative cell number of wild type or *RB1*^*−/−*^ RPE1 cells treated with vehicle (DMSO), 2DDG and OPDLS (*p < 0.05). *n* = 3 independent experiments. Error bars represent standard deviations from these replicates.

Although this data provides molecular details of how pRB-depletion alters cells, we wanted to determine which of these changes are passenger events and which sustain the growth of cells in vivo. To bypass the high levels of functional redundancy in human cells, we used an established E2f1-RNAi *Drosophila* eye model to determine how the depletion of homologs of each of these upregulated proteins modified E2f1-RNAi phenotypes. Using the Glass Multiple Reporter (GMR) GAL4 driver, we ectopically expressed a UAS-E2f1 construct to deplete E2f1. GMR-E2f1-RNAi alters the progression of the morphogenic furrow across the developing eye disc, resulting in aberrant proliferation, metabolism^5^, and apoptosis^24^. These effects have previously been shown to be rescued or exacerbated by changes in pRb, E2f, and Cdk family members^24^. We selected this system, as GMR-mediated depletion of E2f1 results in a strong overgrowth phenotype (Fig. 6a) that is highly modifiable, reproducible, and enables straight-forward screening. In contrast, GMR-Rbf1-RNAi results in a rough eye phenotype that is harder to screen. Importantly, screens conducted in both settings, produce highly similar results. We then tested how RNAi-mediate depletion of the *Drosophila* homologs of 354 upregulated proteins in pRB-depleted RPE1 cells affected the GMR-Gal4/UAS-E2f1-RNAi rough eye phenotype. From this analysis, we identified 28 genes that when knocked down were able to suppress the E2f1-RNAi phenotype (Fig. 6b). Similarly, we found 30 genes that when depleted could enhance or expand the area of the eye affected by the E2f1-RNAi phenotype (Fig. 6c). Each of these modifiers were independently test against control GFP-RNAi lines and found to have no effect on normal eye development. Analysis of these results identified a number of metabolic and cell cycle genes as important for E2f1-RNAi phenotype (Fig. 6d). Pathway analysis of enhancers of the E2f1-RNAi phenotypes was linked to TNF and EGFR signaling and the p53 transcription factor (Supplementary Fig. 5A). In contrast, the suppressors were functionally linked to HSP90 and CCND1 activity (Supplementary Fig. 5B). To evaluate how these processes affect the growth of *RB1*^*−/−*^ RPE1 human cells, we used small molecular inhibitors that alter glucose (2-Deoxy-D-Glucose, 2DDG) or serine (O-phospho-DL-serine, OPDLS) metabolism. These pathways were selected as glucose pathways manipulations exacerbated *Drosophila* E2f1-RNAi phenotypes, in contrast, changes to serine metabolism suppressed the phenotypes. Wild type and *RB1*^*−/−*^ RPE1 cells were treated with vehicle control (DMSO), 2DDG, or OPDLS for 3 days and the relative number measured. As shown in Fig. 6e, *RB1*^*−/−*^ cells are highly sensitive to OPDLS treatment, while being resistant to 2DDG. This data shows that some of the proteomic changes in pRB-depleted cells have conserved roles in maintaining cells with de-regulated E2F activity in *Drosophila* and human cells.

## Discussion

Despite the great clinical need, finding therapeutic strategies to target pRB-depleted cancer cells has proved to be a difficult challenge. A major obstacle is that there is surprisingly little information about the ways that the inactivation of pRB changes the molecular characteristics of non-tumorigenic human cells. Previous studies, using *Rb1* mutant mouse tissue, have shown that the current, transcription-centric view of the effects of pRB loss is incomplete, but proteomic profiling of heterogeneous mouse tissues provided only a low-resolution picture of the changes caused by the mutation of *Rb1*^23^. Here, we report quantitative measurement of changes to the metabolome, transcriptome, and proteome that occurred when pRB is removed from a homogenous diploid non-transformed human cell line. Collectively, this data provides a detailed picture of the pathways affected by pRB loss. Although pRB is often categorized in simple terms as a cell cycle protein or a transcriptional repressor, this data builds on other studies to illuminate the way that pRB loss has broad cellular consequences that impact diverse cellular pathways.

We highlight three features of pRB-depleted cells that are evident when the different sets profiles are viewed together. First, there are many examples where changes seen in one type of data are reinforced by changes in another. For example, pRB-depletion increased the levels of a suite of enzymes involved in purine and pyrimidine synthesis. These changes were accompanied by increases in the levels of nucleotides and nucleosides, suggesting a concerted increase in their synthesis. As pRB-depleted cells and pRB-deficient cancer cells have altered DNA replication rates, it is likely that higher nucleoside and nucleotide generation is essential to sustain these processes. In a similar way, systematic increases in the levels of glycolytic enzymes and the levels of the associated metabolites clearly demonstrate an upregulation of glycolytic pathway in pRB-depleted cells. These results strongly support recent observations of reduced mitochondrial function in pRB-depleted cells. Our data and that from a number of other groups strongly suggest that pRB-loss begins the metabolic reprogramming of cells and mitochondrial mis-regulation. After just 6 days of pRB-depletion, we find that multiple mitochondrial pathways including key metabolic and nucleoside producing reactions are directly altered. Research from a number of different models have demonstrated that pRB-depletion results in mitochondrial changes and our data suggest that these changes are apparent within days of pRB-loss. More work is needed in additional cell lines and at different time points to understand how metabolic flux and mitochondrial activity are altered by pRB-deficiency. However, these studies provide a platform expanding these important questions into different models and cancer contexts.

Second, each type of profiling provides a different perspective on the changes in a pRB-depleted cell. In part, this is because each profiling approach has limitations that make it easier to detect changes in some processes, but not others. For example, low abundance proteins are difficult to quantify by mass spectrometry, and the insights provided by metabolic profiling are limited by the number and variety of standards used to identify peaks. Transcription profiles of RB-depleted cells emphasize changes in E2F targets, proteome profiles highlight changes in mitochondrial proteins and other pathways, while the metabolic profiles show changes in glucose metabolism and nucleotide synthesis. Each of these changes are genuine consequences of pRB inactivation and it is the integration of the different phenotypes that gives the fullest picture of the effects of pRB loss.

Third, perhaps the most curious and unexpected insights stem from differences between the datasets. It is important to appreciate that while some of the transcriptional changes resulting from pRB-depletion are accompanied by a measurable change in protein levels, many do not. Indeed of the most striking features of this analysis are the differences in pathways changed at the mRNA and protein level. Our Gene Ontology profiling of each dataset found limited overlap between mRNA and protein changes and merging this information suggested that pRB-loss results in a complex transcriptional and proteomic response. In particular, the direct E2F target genes that we had hypothesized to be increased at the mRNA and protein levels, can actually be sub-divided into multiple different RNA: protein categories. The majority of the traditional E2F target genes are transcriptionally upregulated but only a subset these genes are also increased at the protein levels. These observations suggest that many E2F targets genes are subject to post-transcriptional or translational control. Interestingly, the factors that are strongly elevated at the mRNA but not the protein level show longer coding sequences, and have longer 5′UTRs and 3′UTRs (Supplementary Fig. 5C–E). These results show that post-transcriptional regulation of E2F-induced mRNAs is important, and likely buffers the effects of E2F activation on the levels of protein required for cell proliferation and apoptosis.

Finally, by testing which of these upregulated pathways are important for the growth and survival of *Drosophila* models and *RB1*^*−/−*^ human cells, our analysis has identified important proteomics events. Perhaps unsurprisingly, we find that cell cycle components act to both enhance and suppress in vivo phenotypes as these cells clearly have altered cell cycles, proliferation, and differentiation programs. The largest and most striking collection of genes that function to drive these phenotypes are metabolic, and have diverse roles in nucleotide generation and TCA cycle progression. This is likely due to elevated metabolic demands of the cells and the need for high levels of building blocks necessary for DNA replication, DNA repair, and growth. This data from *Drosophila* models accurately represents metabolic sensitivities as inhibiting these pathways in *RB1*^*−/−*^ human cells, significantly slows the growth and triggers widespread apoptosis. Many of these change make sense based on how we understand pRB-depletion alters the growth profile of human cells, however the role of many of these upregulated but essential genes remains untested or unknown. Key factors regulating Ribosome assembly, Vacuole transport and cell adhesion are very strong modifiers of the E2f1-RNAi phenotype, suggesting that widespread cellular changes are important for responding to both direct and indirect changes driven by pRB-depletion and E2F-deregulation.

Given that pRB and E2F are involved in the temporal control of gene expression, we appreciate that the absence of a measurable difference in the overall levels of a protein in a population of cells does not necessarily mean that the transcriptional changes are unimportant. However, we note that the data described in these experiments show the depletion of pRB does lead to statistically significant changes in levels of hundreds of proteins, and many of these changes are not evident in the transcript data. The proteins altered in level by the depletion of pRB function in diverse cellular processes and we hope that further studies of these changes may reveal features of the pRB-depleted state that are vulnerabilities and that can be targeted therapeutically.

## Methods

### Antibodies

pRB antibody (1:1000): Abcam (ab181616), ATP5E (A-11) (1:500): Santa Cruz Biotechnology, Inc. (sc-393695), TrpRS (C-7) (WARS) (1:500): Santa Cruz Biotechnology, Inc. (sc-374401), ADPGK (AA40) (1:500): Santa Cruz Biotechnology, Inc. (sc-100751), β-Actin (1:1000): Cell Signaling Technology (#4967), CS (1:1000): Sigma, SAB 2701077, p-pRB (Ser807/811) (1:1000): CST (#8516 S), RBL1 (1:500): Santa Cruz (sc-250), RBL2 (1:500): Santa Cruz (sc-374521), S6 (1:1000): CST (#2217) and S6K Ser235/236 (1:1000): CST (#4858).

### Cell culture

RPE1, BJ, and murine 3T3 cells were grown DMEM containing 5% (tetracycline free) FBS and Glutamine under standard culturing conditions. RPE1 and BJ cells are both from ATCC, and were confirmed using Short Tandem Repeat (STR) profiling. The 3T3 cells were provided by the Dyson group and confirmed by STR profiling. Firefly Luciferase and RB depletion cells were grown in the media described above supplemented with 4 µg/ml of Doxycycline for 3 days before being split and grown in new DOX-supplemented DMEM/FBS for a further 3 days. Non-DOX-treated cells were grown using the same protocol without the addition of DOX.

### Plasmids and generation of pINDUCER11 stable lines

The pINDUCER11 plasmids targeting Firefly Luciferase and pRB were kind gifts from Steven Elledge^28^. Virus was generated using standard lentiviral protocols. To produce stable lines, RPE1 cells were independently infected with lentivirus containing either pRB or Firefly Luciferase targeting pINDCUER11. Three days post-infection, cells were trypsinized and sorted via live Fluorescence-activated cell sorting (FACS) for GFP positivity. Strongly GFP-positive cells were collected and used to generate independent pooled stable cell lines.

### FACS

Cells were labeled briefly with 20 µM Edu (Click Chemistry Tools) for 2 h, incubated at 37 °C, fixed with 3.7% formaldehyde for 15 min, permeabilized in 0.5% Triton-X 100 in PBS. Each step was followed by a wash using 3% BSA in PBS, centrifuged on a table-top centrifuge at 2000g for 1 min. The cells labeled with Edu were incubated in the Click-IT reaction mix (100 mM Tris-Cl (pH7.5), 3 mM CuSO_4_, 50 mM Ascorbic Acid, 2.5 µM Alexa-647 Azide) for 30 min in dark. The cells in specific cell cycle stages were detected using 3 µM (892 ng/ml) DAPI in PBS. All samples were analyzed in triplicates using BD LSR-Fortessa flow cytometer and BD FACS-Diva 8.0.1 software.

### RNA sequencing and differential expression analysis

Total RNA was extracted from cells using Trizol. Total RNA Sequencing libraries were prepped using the TruSeq Stranded Total RNA kit including rRNA depletion with Ribo Zero Gold kit (Illumina). 250 ng of RNA were used for library preparation following the supplier’s instructions, including 10 cycles of PCR amplification. The quality of the library was assessed by TapeStation (Agilent) using the high sensitivity D1000 reagents. Library was quantified with Qubit (Thermofisher) and paired-end sequenced (41 cycles each way) using Nextseq 500 High output v2 kit (Illumina) on the NextSeq500 at the MGH Cancer Center.

Files containing RNA sequencing reads were adaptor and quality-trimmed using TrimGalore-0.6.0. Bowtie2 (version 2.2.9) was used to removing contaminating reads from ribosomal RNA and transfer RNA^37–39^. The trimmed and contamination-filtered reads were mapped to the hg38 genome (GENCODE Release 31) using STAR aligner version 2.5.2a and a counts matrix was obtained using the ‘Gene Counts’ option^40^. The DESeq2 (version 1.22.2) package was used to perform a differential expression analysis using R version 3.5.3^41^. The Ensembl IDs were converted to gene symbols and names using the org.Hs.eg.db package (version 3.7.0). FPKMs were calculated using the fpkm function from DESeq2 using lengths determined via the feature Counts function in the Rsubread package (version 1.32.4) using R version 3.5.0^42^. The data are available at GEO accession number: GSE140383.

### Gene feature length and codon analysis

Gene feature lengths and amino acids compositions were extracted from the RefSeq hg19 annotation, and averaged per gene in case of multiple isoforms. Enrichment/depletion of amino acids are calculated as log2 of the average percentage of the amino acid in the RuPn or RnPu set divided by the average percentage of the amino acids in all genes set. The Kolmogorov-Smirnov test was used to calculate *p*-values.

### Proteomics

A 10 cm dish containing 85–90% confluent RPE1 cells containing different pINDUCER11 construct +/- DOX were collected in duplicate for proteomics. Multiplexed quantitative mass spectrometry-based proteome mappings were done in duplicate using TMT-10 plex reagents and the SPS-MS3 method on an Orbitrap Fusion mass spectrometer (Thermo Scientific)^31,43^. As described previously^43^, disulfide bonds were reduced, free thiols were alkylated with iodoacetamide; proteins were purified by MeOH/CHCl_3_ precipitation and digested with Lys-C and trypsin, and peptides were labeled with TMT-10plex reagents (Thermo Scientific)^31^. Labeled peptide mixtures were pooled and fractionated by basic reversed-phase HPLC as described previously^43^. Twelve fractions were analyzed by multiplexed quantitative proteomics performed using the Simultaneous Precursor Selection (SPS) based MS3 method on an Orbitrap Fusion mass spectrometer (Thermo Fisher Scientific) coupled to an Easy-nLC 1000 (Thermo Fisher Scientific) with auto sampler^30–32^. Peptides were separated on a micro capillary column (inner diameter, 100 µm; outer diameter, 360 µm; length, 30 cm, GP-C18, 1.8 µm, 120 Å, Sepax Technologies). Peptides were eluted with a linear gradient from 11 to 30 % ACN in 0.125 % formic acid over 165 min at a flow rate of 300 nL/min. The Orbitrap Fusion was operated in data-dependent mode, with a survey scan performed over an *m/z* range of 500–1200 in the Orbitrap with a resolution of 6 × 10^4^, automatic gain control (AGC) of 5 × 10^5^, and a maximum injection time of 100 ms. The most abundant ions detected in the survey scan were subjected to MS2 and MS3 experiments to be acquired in a 5 sec experimental cycle. For MS2 analysis, doubly charged ions were selected from an *m/z* range of 600–1200, and triply and quadruply charged ions from an *m/z* range of 500–1200. The ion intensity threshold was set to 5 × 10^4^ and the isolation window to 0.5 *m/z*. Peptides were isolated using the quadrupole and fragmented using CID at 30 % normalized collision energy at the rapid scan rate using an AGC target of 1 × 10^4^ and a maximum ion injection time of 35 ms. MS3 analysis was performed using synchronous precursor selection (SPS)^30,31^. Up to 10 MS2 fragment ions were simultaneously isolated and subjected to MS3 analysis with an isolation window of 2.5 *m/z* and HCD fragmentation at 55 % normalized collision energy. MS3 spectra were acquired at a resolution of 6 × 10^4^ with an AGC target of 1 × 10^5^ and a maximum ion injection time of 100 ms. The lowest *m/z* for the MS3 scans was set to 110. An identical bridge sample from pooling all tryptic digests was added to each of the three TMT-pools analyzed for this study to allow the comparison of proteome profiles across all samples^32^. MS2 spectra were assigned using a SEQUEST-based proteomics analysis platform^44^. The protein sequence database for matching the MS2 spectra was based on the human uniprot protein sequence database. Peptide and protein assignments were filtered to a false discovery rate of < 1 % employing the target-decoy database search strategy^45^ and using linear discriminant analysis and posterior error histogram sorting^44^. Peptides with sequences contained in more than one protein sequence from the UniProt database were assigned to the protein with most matching peptides^44^. We extracted TMT reporter ion intensities as those of the most intense ions within a 0.03 and the window around the predicted reporter ion intensities in the collected MS3 spectra. Only MS3 with an average signal-to-noise value of larger than 20 per reporter ion as well as with an isolation specificity^30^ of larger than 0.75 were considered for quantification. A two-step normalization of the protein TMT-intensities was performed by first normalizing the protein intensities over all acquired TMT channels for each protein based on the median average protein intensity calculated for all proteins. To correct for slight mixing errors of the peptide mixture from each sample a median of the normalized intensities was calculated from all protein intensities in each TMT channel and the protein intensities were normalized to the median value of these median intensities. Mass spectrometry RAW data are accessible through the MassIVE data repository (massive.ucsd.edu) under the accession number MSV000084736.

### Proteomics analysis

Differential protein expression between pRB+ and pRB-depleted proteomes was calculated using a T-test. The Benjamini-Hochberg multiple hypothesis correction was applied to calculate corrected *p*-values (FDR). Differential expression of proteins between pRB+ and pRB− samples was considered significant with an FDR < 10% and a minimum absolute log_2_ FC < −0.5 and FC > 0.5. To generate pRB-loss data, we first calculate protein changes in FF-DOX and FF+DOX samples as this represents non-specific or DOX-mediated changes in the proteome. These proteins were removed from both the RB25 and RB26 data. We then compared the proteome of FF-DOX, RB-25-DOX, and RB26-DOX and found no statistically significant differences. These normalized data were then used to identify proteomic changes after pRB-loss from RB25 and RB26 separately.

### Harvesting human cells for LC-MS/MS

To harvest intracellular metabolites, wells were washed once with 150 mM sodium chloride solution. Wells were aspirated and intracellular metabolites from cells on dry ice by scraping cells from each well into 500uL of chilled Acetonitrile/methanol/water (40/40/20) (Sigma, ACS grade), samples were quickly vortexed and placed on dry ice to freeze. A series of 3 freeze/thaw cycles were performed. The cellular lysate was clarified at 4 °C with a spin of 21k × *g* for 10 min. Clarified lysate was transferred and then lyophilized and snap frozen in liquid nitrogen. Prior to mass spectrometry analysis, samples were re-suspended using 20 µL HPLC grade water.

### Metabolite LC-MS/MS analysis

LC-MS/MS analysis was done as previously described^5,46^. Briefly, analysis was performed on a 5500 QTRAP hybrid triple quadrupole mass spectrometer (AB/SCIEX) coupled to a Prominence UFLC HPLC system (Shimadzu). For analyses of metabolite pools 10 µL of each sample was injected and analysis was done using selected reaction monitoring (SRM) of a total of 258 endogenous water-soluble metabolites. As some metabolites were targeted in both (+) and (−) ion mode, a total of 289 SRM transitions due to ion mode switching were analyzed. For 13 C labeled experiments, specific SRMs were constructed based on predicted 13 C incorporation patterns. Desired ions were produced using an ESI voltage of +4900 V in (+) ion mode and −4500 V in (−) ion mode. Between 9 and 12 data points were acquired per detected metabolite using a dwell time of 3 ms per SRM transition and a total cycle time of 1.55 s. Sample delivery to the MS was done under normal phase chromatography using a 4.6 mm i.d × 10 cm Amide Xbridge HILIC column (Waters Corp.) at 300 ml/min. Gradients were run starting from 85% buffer B (HPLC grade acetonitrile) to 42% B from 0 to 5 min; 42% B to 0% B from 5 to 16 min; 0% B was held from 16 to 24 min; 0% B to 85% B from 24 to 25 min; 85% B was held for 7 min to re-equilibrate the column. Buffer A was comprised of 20 mM ammonium hydroxide/20 mM ammonium acetate (pH = 9.0) in water: acetonitrile (95:5). Peak areas from the total ion current for each metabolite SRM transition were integrated using MultiQuant v2.0 software (AB/SCIEX).

### Glycolysis assay

Glycolysis Assay (Abcam #ab197244) was performed to measure extra-cellular acidification as a read-out for lactate metabolite. 5 × 10^4^ cells were seeded per sample on a 96-well black plate and incubated in a 37 °C, 5% CO2 incubator overnight for the cells to attach. The next day, the glycolysis assay was performed as mentioned in the manufacturer protocol. The fluorescent reading was obtained on SpectraMax microplate reader using excitation and emission wavelengths of Ex/Em = 380/615 nm.

### Gene ontology analysis

Differentially expressed genes and proteins were measured for enriched gene ontology terms using DAVID website tool (https://david.ncifcrf.gov/)^47–49^. Genes and proteins with statistically significant level changes were mapped to different gene ontology pathways using stringent program settings.

### Ingenuity pathway analysis (IPA)

Core expression analysis was performed using the commercial software Ingenuity Pathway Analysis (Ingenuity™ Systems; IPA; http://www.ingenuity.com) tool. The analysis performed was based on log ratio of protein expression in pRB-KD/WT. The input list was normalized with the reference gene set from Ingenuity Knowledge Base (Genes only). Graphical representations of the networks containing direct relationships were generated for both enhancers and suppressors obtained from the fly screen.

### Chromatin immunoprecipitation (ChIP)

ChIP was performed using protocols and reagents from the Simple Chip Enzymatic Chromatin IP Kit (CS 9003). Proliferating RPE1 cells were cross-linked in 1% formaldehyde, rocking for 10 min at room temperature. Crosslinking was quenched with glycine at a final concentration of 0.125 M, rocking at room temperature for 5 min. Plates were washed twice with ice cold PBS, scraped into a new tube, and pelleted by centrifugation at 350 *g* for 5 min. The supernatant was removed and pellet was processed according to the manufacturer’s protocols. Chromatin was allowed to incubate with 5 µg of either mouse anti-E2F1 (EMD Millipore 05–379) or normal mouse IgG (Santa Cruz) at 4 °C overnight with rotation. 8 equivalents of beads were added to immunoprecipitate the antibody-bound chromatin. After elution and reversal of crosslinks, chromatin was purified by precipitation with ethanol overnight at −20 °C. Chromatin concentration was determined using a Qubit Fluorimeter and a Qubit dsDNA High Sensitivity Kit (Thermo Fisher Q32854). Real-time qPCR was performed using Fast Start Universal SYBR Green Master Mix with Rox (Roche) using the Applied Biosystems Step One Plus system. 100 ng of input DNA and 12.5 ng of IP DNA were added to each well. Primer sequences are list below (Table 1). Each ChIP was conducted in biological and technical triplicates and the data included within the paper represent means and standard deviations of these reactions.

### ChIP primers

**Table 1:** Primers used for ChIP in this study.

hMcm3p ChIP F	GCGCGAAAACTTCCGAACTC
hMcm3p ChIP R	CAGTCGCTAGTCCGACCTCA
hAPCp ChIP F	TGGATGAGGCCCTCTGTTAGA
hAPCp ChIP R	GCAGCACCCTGTGAACATCT
hADSLp ChIP F	TGTAGCGCGGCTAAGTAACG
hADSLp ChIP R	GACTTCCTTCACGCGGTCTC
hADSSp ChIP F	CCACTCCCGTGCAAACCTC
hADSSp ChIP R	TTGAAAGCGAGAGGTAGAGCG
hTIP1p ChIP F	CTTCGTTGGGGGAAACTGGA
hTPI1p ChIP R	CGAGGGCTTACCGGTGTC
hLDHA ChIP F	GTGCATTCCCGGTACGGTAG
hLDHA ChIP R	GAAAGCGGCTCCTACACCTC
hLDHB ChIP F	TCTGTAACAGTCGTGCGGAG
hLDHB ChIP R	AGGCGGTACTCGGATTTCCA
hOASL ChIP F	CAGAAACTCCTCCACGGTCC
hOASL ChIP R	ATATATCTGCCCAGCAGCGG
hWIBGPYM1p ChIP F	ACTCGCCAATAATCCGGTCC
hWIBGPYM1p ChIP R	AACAGTTGCTGAAATGGGCG
hFTDSJD2p ChIP F	AAAGGCCCACCTCACCTAGA
hFTDSJD2p ChIP R	AGGGACCTCAGTCTTCCCTC
hSOD1p ChIP F	TAAAGTAGTCGCGGAGACGG
hSOD1p ChIP R	TTCGTCGCCATAACTCGCTA
hCTPS1p ChIP F	GTCGCTGACGGGAGGATCT
hCTPS1p ChIP R	CGCCCCTGATTGGTAGGAG
hALDOA N ChIP F	TGGCCGGAGATACATCCAAG
hALDOA N ChIP R	CCTCATCAGATGCCGGACG
hPPAT R ChIP F	TGAGTAACAGGGATCCGGGC
hPPAT R ChIP R	CGAGGCAGTAAAGTTCGGGG
hIMPDH2 ChIP F	CTGGGCCGCGCCAATATAAA
hIMPDH2 ChIP R	ATACGCATGCGCTGTTTCTTC
hALDOA H ChIP F2	TGGGGAGGGATCGTGTTCTC
hALDOA H ChIP R2	TTTCCTTTACGAGGAGCGGG

### RNA isolation, cDNA synthesis, and quantitative RT-PCR

RNA was harvested from RPE1 cells, Wild type, and *Rb1*^*−/−*^ MEFs using the Qiagen RNaeasy minikit as per the manufacturer’s instruction. From these extractions, 400 ng of RNA from each cell line was used to generated cDNA was prepared using the High Capacity cDNA Reverse Transcription kit (Applied Biosystems) according to the manufacturer’s protocol. The expression of the specified target genes was quantified by q-RT-PCR using Fast Start Universal SYBR Green Master with ROX (Roche) and was normalized to the expression of β-actin and normal samples. The primers used for the qRT-PCR reactions are list below and each reaction was conducted in biological and technical triplicates and the data presented represents means and standard deviations from these reactions. All q-RT-PCR experiments were performed using Roche SYBR green master mix under the following cycling conditions: Initial denaturation – 95 C (10 mins); 95 C (15 s), 60 C (1 min) for 40 cycles; 95 C (15 s), 60 C (1 min), 95 C (15 s) for melt curve.

### RT-PCR

RNA was extracted using Trizol reagent and 250 ng of RNA was used to prepare cDNA. Reverse transcription was performed as mentioned in the manufacturer protocol using Applied Biosystems High-Capacity cDNA Reverse Transcription Kit. The relative expression of the targets mentioned was calculated using Ct values obtained from performing q-RT-PCR using Fast Start Universal SYBR Green Master with ROX (Roche) and was normalized to the control sample. All experiments were performed in at least triplicates, unless mentioned otherwise. Custom primers were designed (Table 2).

**Table 2:** Primers used for RT-PCR in this study.

Gene	Primers	
	Forward	Reverse
RB1	GAACATCGAATCATGGAATCCCT	AGAGGACAAGCAGATTCAAGGTGAT
BAD	CTCCGGAGGATGAGTGACGA	CACCAGGACTGGAAGACTCG
BID	AAGAAGGTGGCCAGTCACAC	AAGAAGGTGGCCAGTCACAC
BCL2	GGGGTCATGTGTGTGGAGAG	ACCTACCCAGCCTCCGTTAT
BAK1	TCATCGGGGACGACATCAAC	CAAACAGGCTGGTGGCAATC
CASP3	GGATGGCTCCTGGTTCATCC	TCTGTTGCCACCTTTCGGTT
CASP8	GAGCCTGAGAGAGCGATGTC	AGGCTGAGGCATCTGTTTCC
ARL14	CAAACCAAACAAGCCCAAGT	CCTCCAACATCCCAGACTGT
SERPINA9	CTCACTGACTGTTCCCAGCA	ATGGAGGGGTTGGAGAAATC
IL18	AAGATGGCTGCTGAACCAGT	TCTGATTCCAGGTTTTCATCATCT
TPI	TTCGGCAACCTGAACGACTC	TTGGGGAAAGCGGTAGGATG
PGK1	GCTGGACAAGCTGGACGTTA	TGGGACAGCAGCCTTAATCC
ORC1	CCCTATCAGTGGGGGACAGA	ATGGGGAGTAGAGGTCGCTT
SMC2	TCAGCCAGATGTATTGCACCA	CACATGAACGTTGTCAGGGC
SMC4	CCTGTTGTCATGCACTGGACT	TCGGTCATCTTTTTCGCCCA
MCM4	TGTTTGCTCACAATGATCTCG	CGAATAGGCACAGCTCGATA
E2F1	ACAAGGCCCGATCGATGTTT	GAGTCAGTGGCCCTGTTCTC
CENPE	GATTTGGATGAATTTGAGGCTCT	ACTTCTGCATGCTTAACTAAATTCT
ACTIN	TCACCCACACTGTGCCCATCTACG	CAGCGGAACCGCTCATTGCCAATG

### Comparison between RPE1, murine, and SCLC proteomics

We compared our proteomics results from the doxycycline-dependent inducible knockdown of RB (*p* < 0.05 and log2 FC > 0.3785) to previously published proteomics results from murine lung and colon samples from a pRB loss study^23^, as well as a previously published small cell lung cancer study (SCLC)^36^. We overlapped significant results from our doxycycline results with lung or colon proteins with a log FC > 0.3785 and with the same direction fold-change. These results in turn were overlapped with the previously identified SCLC-specific proteins.

### Retinoblastoma protein dataset

The dataset from the proteomic profiles of seven Retinoblastoma tumors and three normal retinal tissues was downloaded^35^ and the average of each protein level from the tumors normalized to retinal controls^35^. We overlapped significant results from our doxycycline results with proteins with a log FC > 0.3785 and with the same direction fold-change. Protein changes in Retinoblastoma tumors were normalized to proteins levels in control Retinal tissue.

### Retinoblastoma RNA dataset

The RNA profiles of Retinoblastoma tumor and normal retinal samples were downloaded^34^. Files containing RNA sequencing reads were adaptor and quality-trimmed using TrimGalore-0.6.0. Bowtie2 (version 2.2.9) was used to removing contaminating reads from ribosomal RNA and transfer RNA^37–39^. The trimmed and contamination-filtered reads were mapped to the hg38 genome (GENCODE Release 31) using STAR aligner version 2.5.2a and a counts matrix was obtained using the ‘Gene Counts’ option^40^. The DESeq2 (version 1.22.2) package was used to perform a differential expression analysis using R version 3.5.3^41^. The Ensembl IDs were converted to gene symbols and names using the org.Hs.eg.db package (version 3.7.0). FPKMs were calculated using the fpkm function from DESeq2 using lengths determined via the feature Counts function in the Rsubread package (version 1.32.4) using R version 3.5.0^42^. RNA changes in Retinoblastoma tumors were normalized to RNA levels in control Retinal tissue.

### Western blotting from retinoblastoma tumors and retinal tissue

The present study was approved by the institutional ethics board and conducted at Medical research foundation and Vision research foundation, Sankara Nethralaya, India with Ethics No. 247-2011-P. Control retinas from cadaveric people were collected from C.U. SHAH eye bank, Sankara Nethralaya. Tumor tissues were collected with informed consent from the parents/guardians of the patient. The Tumor (N = 6) and Retina (N = 3) tissues were lysed in 1X RIPA buffer (Thermo Scientific, Cat no: 89900) along with Protease Inhibitor Cocktail (PIC) (Sigma Aldrich) by sonication followed by centrifugation at 12,000 rpm at 4 °C for 10 min. The supernatant was collected and stored at −80 °C until further use. The concentration of the protein was estimated by Bicinchoninic Acid assay (BCA) method (Pierce BCA protein Assay kit, Cat no: 23225) with varying concentrations of Bovine Serum Albumin (BSA) as standards.

Protein lysates (150 μg) were mixed with approximately 1X sample loading buffer (0.25 M Tris-HCl pH 6.8, 15% SDS, 50% glycerol, 25% β-mercaptoethanol, 0.01% bromophenol blue). The samples were denatured at 95 °C for 10 min and run on 10% Sodium Dodecyl Sulfate - Polyacrylamide gel electrophoresis (SDS-PAGE) at a constant voltage of 100 V for 90 min. The proteins were then transferred onto a nitrocellulose membrane (wet transfer) at 100 V for 60 min. 5% non-fat milk in PBS with 0.01% Tween 20 (PBST) was used as blocking reagent to prevent non-specific binding. After blocking, the membrane was probed with anti-CTPS (Sigma, Cat no: WH0001503 M1) or anti-CS (Sigma, SAB 2701077) primary monoclonal antibody (1:1000 in PBST) and incubated overnight at 4 °C. The membranes were washed thrice with PBST for 10 min at room temperature, subsequently probed with respective HRP conjugated secondary goat Anti-Mouse IgG HRP (Santa Cruz, Cat no: sc-2005) (1:3000 in PBST) and incubated for 2 h at room temperature. After intermittent washes with PBST (3 × 10 min) to remove the unbound secondary antibodies, the membrane was detected using a TMB substrate. Protein lysates probed with anti-β actin primary monoclonal antibody was used as an endogenous control for the Western blotting experiments. Results were compiled as a mean of three independent experiments. The data are presented as the Mean ± SD of at least three independent experiments.

### Drosophila screen and imaging

GMR-G4-UAS-E2f1-RNAi/CyO virgin females were crossed to UAS-RNAi lines targeting the proteins of interest. Each of the UAS-RNAi lines were purchased from the Bloomington Drosophila Resource Center (BDRC). 3 days after the cross was set, the adults were moved to a new vial and then discarded 3 days later. Non-CyO progeny from each cross was independently scored by blinded screeners and at the end of the screen, the scores were merged. All modifiers were then re-crossed to GMR-G4-UAS-E2f1-RNAi/CyO for confirmation, GMR-G4-UAS-GFP-RNAi/CyO to test whether the phenotype was E2f1-RNAi dependent and PTC-G4-UAS-E2f1-RNAi/CyO to determine if each line could modify non-eye E2f1-RNAi phenotypes.

For Scanning Electron Microscopy (SEM), the Drosophila were dehydrated in increasing levels of ethanol before being critical point dried and imaged using the CEMAS SEM facility at the Ohio State University. Multiple flies were imaged for each modifier and representative images selected for inclusion in the paper.

### Cell death assay

5×10^4^ cells were seeded in each well of a 24-well plate for RPE1-WT and RB-KO clones. The cells were incubated overnight at 37 °C, 5% CO_2_ incubator and allowed to attach. 2-Deoxy Glucose (62.5 nM, Sigma #D8375) and O-phospho-DL-serine (250 nM, Sigma #79710) were added to the treatment wells with the corresponding vehicle control. The cells were placed in the 37 °C, 5% CO_2_ incubator for 72 h. Cells were then fixed in the crystal violet fixing solution and stained with crystal violet for visualization.

### Statistics and reproducibility

Statistical analysis of experimental data was conducted using t-tests or ANOVA tests replicates. We define biological replicates as cells harvested from independent experiments, and technical replicates as the number of samples collected in multiple from each biological replicate. All cellular experiments were conducted in biological and technical triplicates. Replicate number and strategy for RNA-seq, proteomics and metabolomics are extensive outlined in those sections. In brief, we used 2 independent shRNAs targeting pRB or controls. Biological replicates were defined as cells expressing each shRNA and technical replicates were generated by conducting the experiment in triplicate.

### Reporting summary

Further information on research design is available in the Nature Research Reporting Summary linked to this article.

## Supplementary information

Supplementary Information

Supplementary Data 1

Supplementary Data 2

Supplementary Data 3

Supplementary Data 4

Supplementary Data 5

Description of Supplementary Files

Reporting Summary

## Data Availability

The RNA-seq data from this work can be found at GEO accession number: GSE140383. The proteomic data from this work can be found at the MassIVE data repository under the accession number: MSV000084736. Source data underlying figures in the main text and supplementary information are presented in Supplementary Data 4–5.

## Code availability

All software and code used to process and analyze the data are described in the “Methods”. These have been previously described in the literature and are common, frequently utilized tools used to measure changes in omics studies.
