# Author Correction: Integrated multi-omics analysis of RB-loss identifies widespread cellular programming and synthetic weaknesses

**DOI:** 10.1038/s42003-021-02708-8

**Published:** 2021-09-30

**Authors:** Swetha Rajasekaran, Jalal Siddiqui, Jessica Rakijas, Brandon Nicolay, Chenyu Lin, Eshan Khan, Rahi Patel, Robert Morris, Emanuel Wyler, Myriam Boukhali, Jayashree Balasubramanyam, R. Ranjith Kumar, Capucine Van Rechem, Christine Vogel, Sailaja V. Elchuri, Markus Landthaler, Benedikt Obermayer, Wilhelm Haas, Nicholas Dyson, Wayne Miles

**Affiliations:** 1 Department of Cancer Biology and Genetics, The Ohio State University, Columbus, OH USA; 2 The Ohio State University Comprehensive Cancer Center, The Ohio State University, Columbus, OH USA; 3 Massachusetts General Hospital Cancer Center, Charlestown, MA USA; 4 Harvard Medical School, Boston, MA USA; 5 Berlin Institute for Medical Systems Biology, Max-Delbrück-Center for Molecular Medicine in the Helmholtz Association, Berlin, Germany; 6 Department of Nanobiotechnology, Vision Research Foundation, Sankara Nethralaya, Chennai, Tamil Nadu India; 7 Department of Pathology, Stanford University, Stanford, CA USA; 8 Center for Genomics and Systems Biology, Department of Biology, New York University, New York, USA; 9 IRI Life Sciences, Institute für Biologie, Humboldt Universität zu Berlin, Berlin, Germany; 10 Core Unit Bioinformatics, Berlin Institute of Health (BIH), Berlin, Germany; 11 Present Address: Agios Pharmaceutical, Cambridge, MA USA

**Keywords:** Tumour-suppressor proteins, Transcriptomics, Cancer metabolism

Correction to: *Communications Biology* 10.1038/s42003-021-02495-2, published online 17 August 2021.

The wrong Supplementary Data 2 was originally published with this article; it has now been replaced with the correct version. The html version of this article has been corrected.

## Supplementary information

Supplementary Data 2

