# Supplementary material for: Integrated multi-omics analysis of RB-loss identifies widespread cellular programming and synthetic weaknesses
Source: Commun Biol. 2021 Aug 17;4:977. doi: 10.1038/s42003-021-02495-2 (PMC8371045; doi:10.1038/s42003-021-02495-2)
Supplement: Supplementary file 1 — Supplementary Information [file 42003_2021_2495_MOESM1_ESM.pdf]

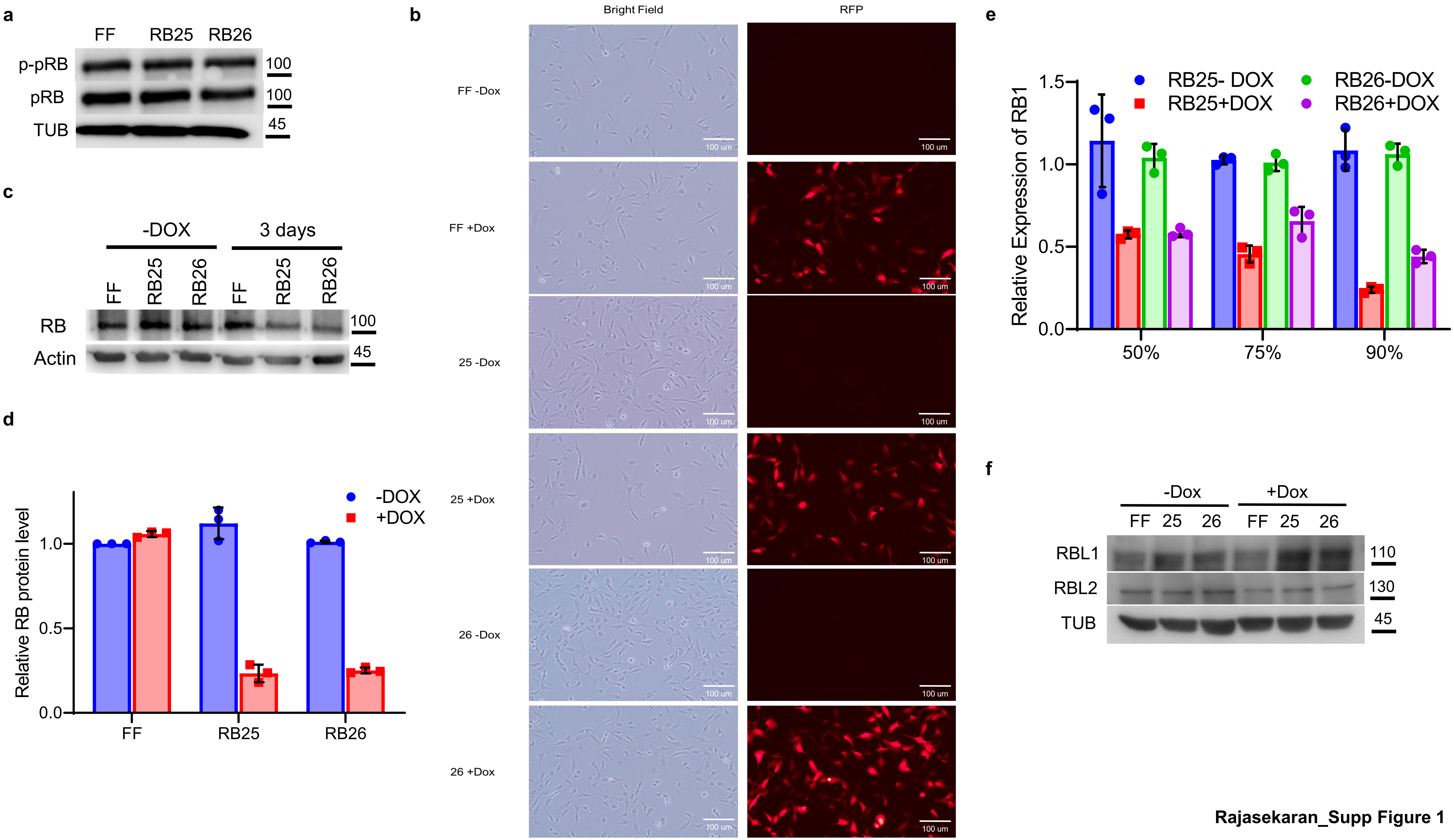

Rajasekaran_Supp Figure 1

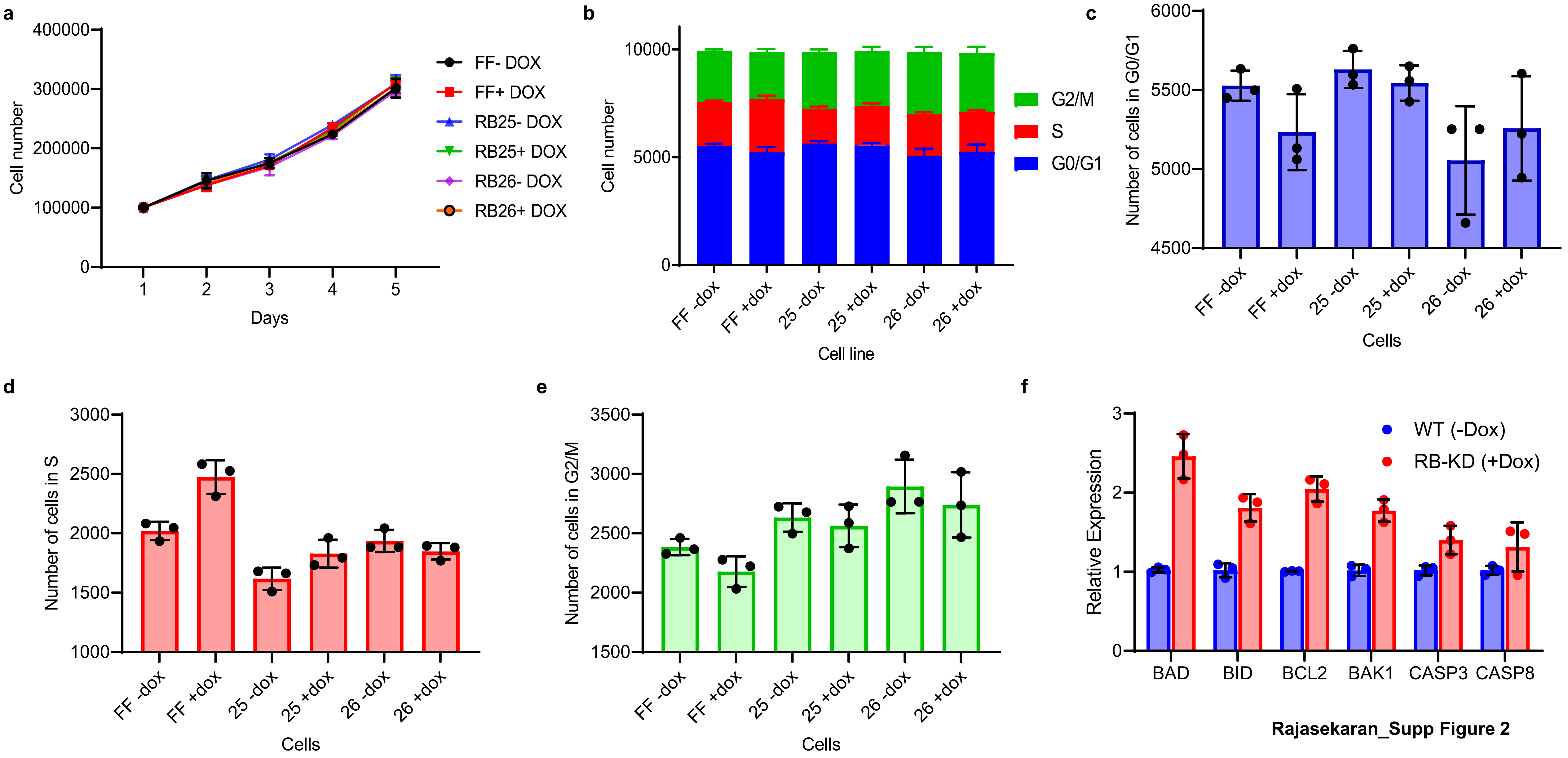

Rajasekaran_Supp Figure 2

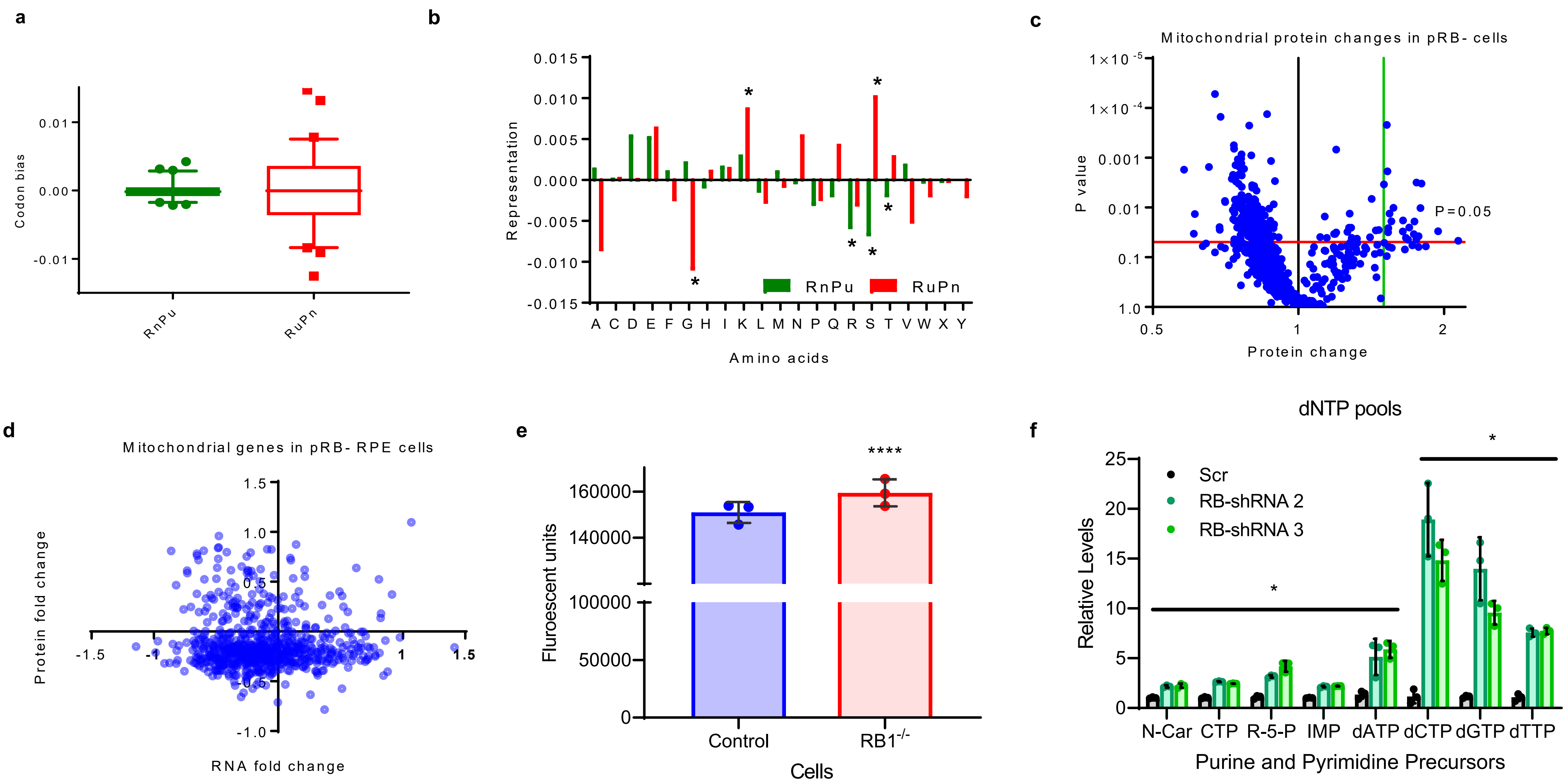

Rajasekaran_Supp Figure 3

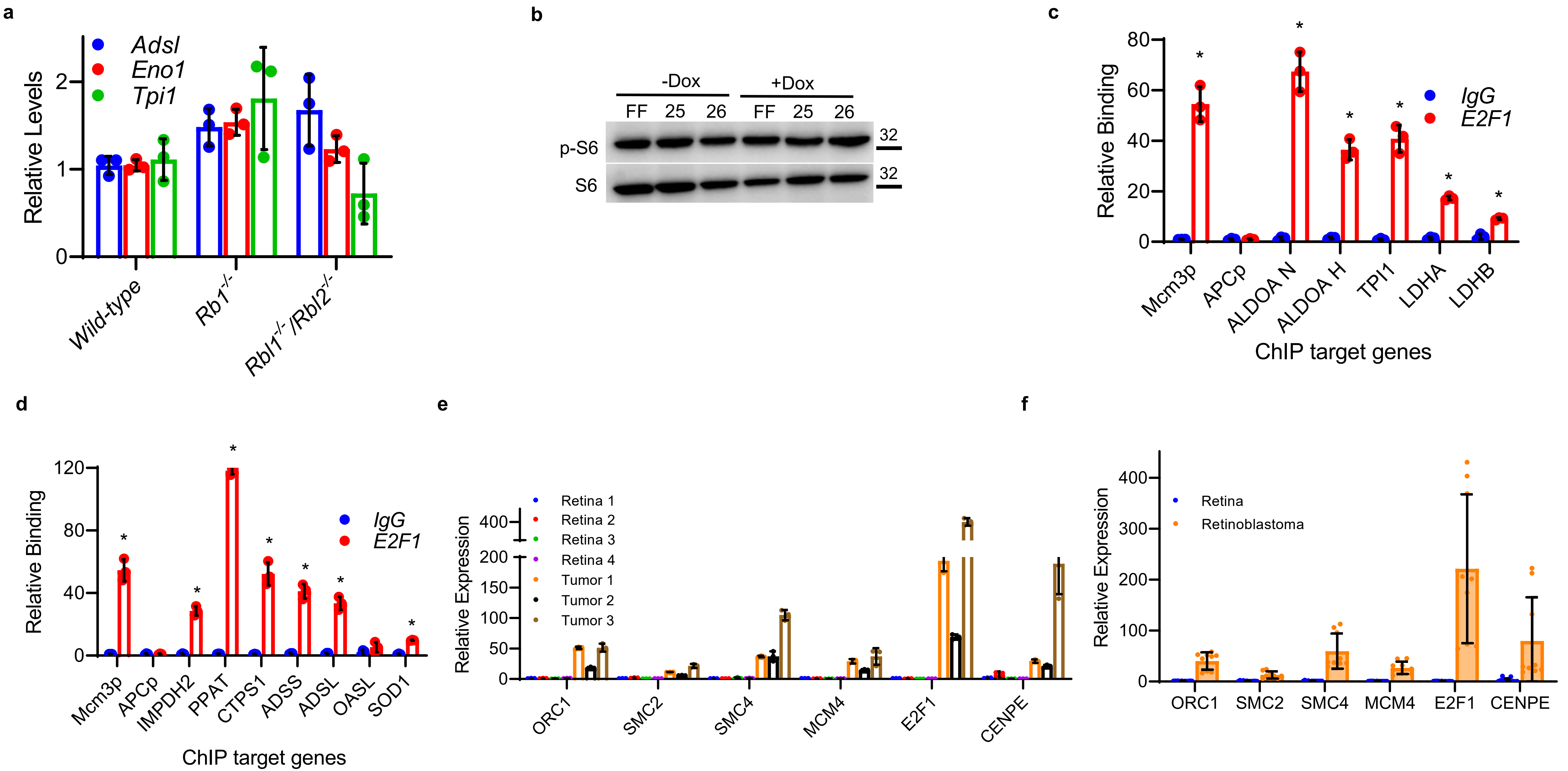

Rajasekaran_Supp Figure 4

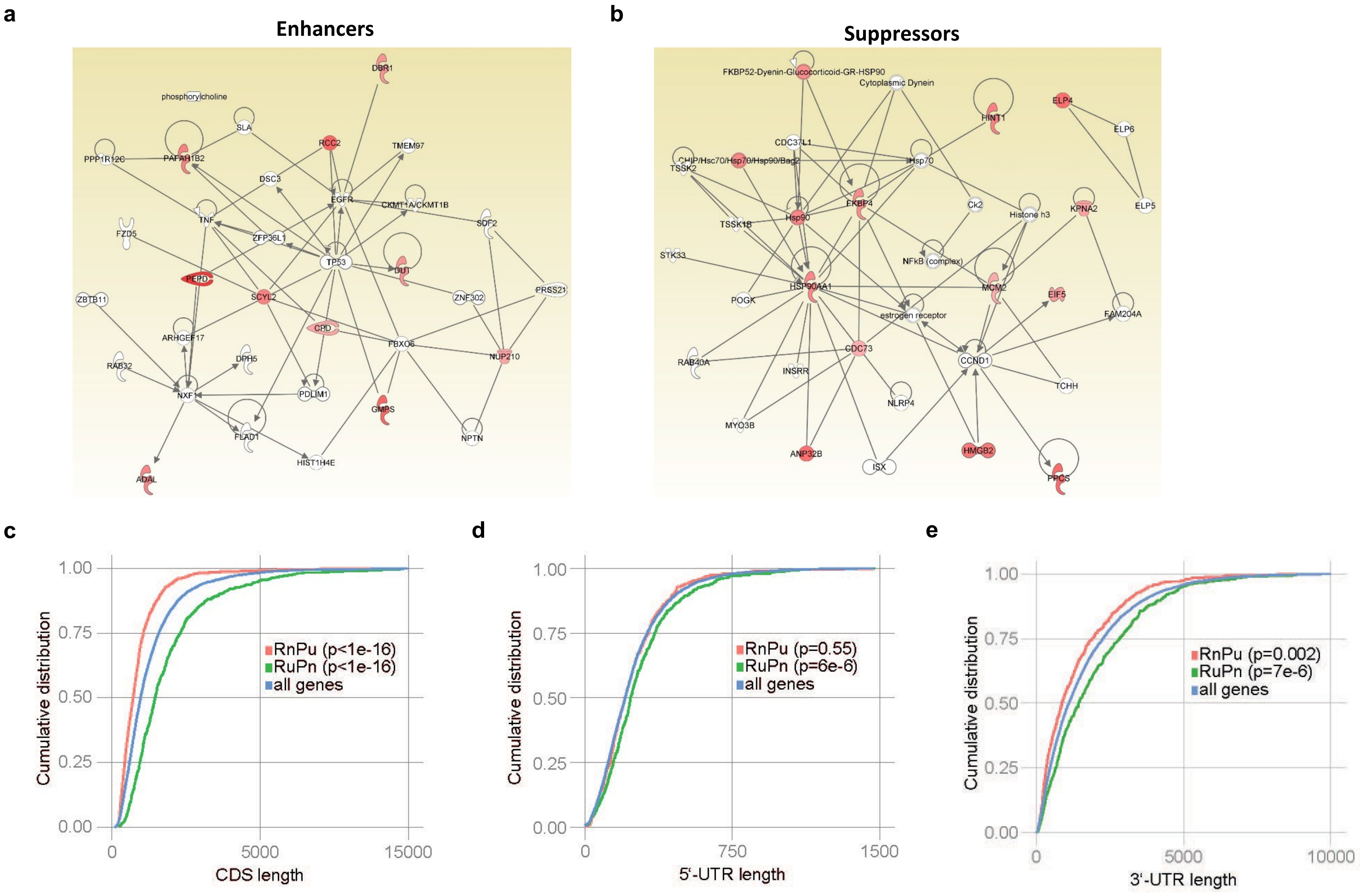

Rajasekaran_Supp Figure 5

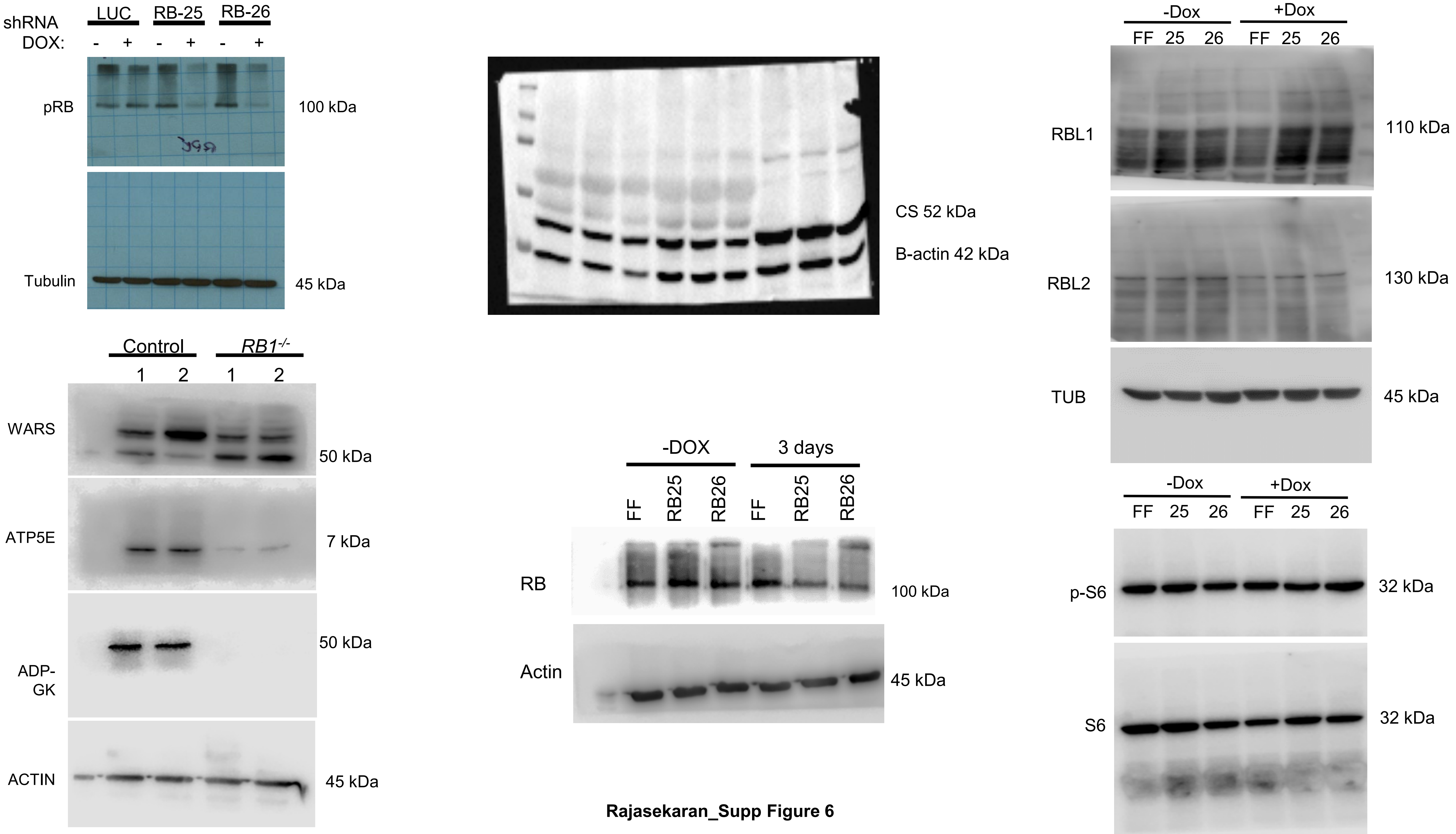

Rajasekaran_Supp Figure 6

Supplementary Figure 1

(A) Western blots of phosphorylated pRB (p-pRB), pRB and TUB from FF, RB25 and RB26 RPE1 cells. **(B)** Bright field and RFP representative images from FF, RB25 and RB26 RPE1 cells treated with (+DOX) or without DOX (-DOX). Bar represents 100µm. **(C)** Western blots of pRB and TUB from FF, RB25 and RB26 RPE1 cells treated with DOX for 3 days or -DOX. **(D)** Relative pRB protein level following 6 days of DOX treatment of FF, RB25 and RB26 cells. n=3 independent experiments. Error bars represent standard deviations from these replicates. **(E)** Relative RB1 expression level following 6 days of DOX treatment at 50%, 75% and 90% confluency. n=3 independent experiments. Error bars represent standard deviations from these replicates. **(F)** Western blots of RBL1, RBL2 and TUB from FF, RB25 and RB26 RPE1 cells +/- DOX treatment.

Supplementary Figure 2

(A) Cell number of FF, RB25 and RB26 cells treated with (+DOX) or without DOX (-DOX) over 5 days. **(B)** Cell cycle distribution of FF, RB25 and RB26 cells with or without DOX treatment for 6 days. **(C)** Number of cells in G0/G1 phase of the cell cycle from FF, RB25 and RB26 cells treated with or without DOX for 6 days. **(D)** Number of cells in S phase of the cell cycle from FF, RB25 and RB26 cells treated with or without DOX for 6 days. **(E)** Number of cells in G2/M phase of the cell cycle from FF, RB25 and RB26 cells treated with or without DOX for 6 days. **(F)** Relative RT-PCR levels of BAD, BID, BCL2, BAK1, CASP3 and CASP8 in wildtype (-DOX) or pRB-depleted (RB-KD (+DOX)) cells. n=3 independent experiments. Error bars represent standard deviations from these replicates.

Supplementary Figure 3

(A) Codon bias in genes upregulated at the RNA level but not protein level (RuPn, red bar) and genes unchanged at the RNA level but have increased protein level (RnPu, green bar) compared to all genes detected in the mass spectrometry assay (n=6363). Error bars represent standard deviations from these replicates. **(B)** Enrichment/depletion of amino acids in the gene set upregulated at the RNA level but not protein level (RuPn, red bars) and genes unchanged at the RNA level but have increased protein level (RnPu, green bars) compared to all genes detected in the mass spectrometry assay (n=6363) (*p<0.0001). **(C)** Protein change of mitochondrial genes from RPE1 cells depleted of pRB (Red line represents p-value of <0.05, and Green line protein fold change higher than 1.5). **(D)** Merged RNA and protein levels changes of mitochondrial genes from RPE1 cells depleted of pRB. **(E)** Fluorescent units measurements of acidification from control and RB1-/- RPE1 cells (****p<0.0001). n=3 independent experiments. Error bars represent

standard deviations from these replicates. **(F)** LCMS data showing purine and pyrimidine metabolite changes from pRB-depleted BJ cells (*p<0.01). n=3 independent experiments. Error bars represent standard deviations from these replicates.

Supplementary Figure 4

(A) Relative RT-PCR of metabolic genes, *Adsl*, *Eno1* and Tpi1, from wild-type, $Rb1^{-/-}$ and $Rbl1^{-/-}$/$Rbl2^{-/-}$ 3T3 cells (*p<0.05). **(B)** Western blots of S6 kinase (S6) and phosphorylated S6 (p-S6) in FF, RB25 and RB26 RPE1 cells with or without DOX treatment. **(C)** Relative binding of E2F1 from Chromatin Immunoprecipitations in RPE1 cells on glucose metabolism genes (*p<0.01). **(D)** Relative binding of E2F1 Chromatin Immunoprecipitation on purine and pyrimidine metabolism genes in RPE1 cells (*p<0.01). **(E)** Relative expression of ORC1, SMC2, SMC4, MCM4, E2F1 and CENPE from individual Retina and Retinoblastoma (Tumor) samples. **(F)** Relative expression of ORC1, SMC2, SMC4, MCM4, E2F1 and CENPE from Retina and Retinoblastoma (Tumor) samples. n=3 independent experiments. Error bars represent standard deviations from these replicates.

Supplementary Figure 5

(A) Ingenuity Pathway Analysis (IPA) of the Enhancers of the GMR-E2f1-RNAi phenotype. Shapes represent different protein types and their cellular roles. **(B)** Ingenuity Pathway Analysis (IPA) of the Suppressors of the GMR-E2f1-RNAi phenotype. Shapes represent different protein types and their cellular roles. **(C)** Cumulative disruption of CDS length in all genes (blue), RNA unchanged: protein upregulated (RnPu) (red) and RNA upregulated: protein unchanged (RuPn) (green). **(D)** Cumulative disruption of 5'UTR length in all genes (blue), RNA unchanged: protein upregulated (RnPu) (red) and RNA upregulated: protein unchanged (RuPn) (green). **(E)** Cumulative disruption of 3'UTR length in all genes (blue), RNA unchanged: protein upregulated (RnPu) (red) and RNA upregulated: protein unchanged (RuPn) (green).

Supplementary Figure 6

Full scans of western blots presented in this manuscript.

Supplementary Data 1: RNA-seq data from pRB-depleted cells.

Supplementary Data 2: Proteomic data from pRB-depleted cells.

Supplementary Data 3: Metabolite data from pRB-depleted cells.

Supplementary Data 4: Source data underlying main figures.

Supplementary Data 5: Source data underlying supplementary figures.
