# Supplementary material for: Integrated multi-omics analysis of RB-loss identifies widespread cellular programming and synthetic weaknesses
Source: Commun Biol. 2021 Aug 17;4:977. doi: 10.1038/s42003-021-02495-2 (PMC8371045; doi:10.1038/s42003-021-02495-2)
Supplement: Supplementary file 7 — Description of Supplementary Files [file 42003_2021_2495_MOESM7_ESM.pdf]

Description of Additional Supplementary Files

File name: Supplementary Data 1

Description: Transcriptional changes in pRB-depleted RPE1 cells.

File name: Supplementary Data 2

Description: Proteomic changes in pRB-depleted RPE1 cells.

File name: Supplementary Data 3

Description: Metabolite changes in pRB-depleted RPE1 cells.
