# Supplementary material for: Integrated multi-omics analysis of RB-loss identifies widespread cellular programming and synthetic weaknesses
Source: Commun Biol. 2021 Aug 17;4:977. doi: 10.1038/s42003-021-02495-2 (PMC8371045; doi:10.1038/s42003-021-02495-2)
Supplement: Supplementary file 8 — Reporting Summary [file 42003_2021_2495_MOESM8_ESM.pdf]

nature research

Corresponding author(s): Wayne Miles

Last updated by author(s): Jul 6, 2021

Reporting Summary

Nature Research wishes to improve the reproducibility of the work that we publish. This form provides structure for consistency and transparency in reporting. For further information on Nature Research policies, see Authors & Referees and the Editorial Policy Checklist.

Statistics

For all statistical analyses, confirm that the following items are present in the figure legend, table legend, main text, or Methods section.

n/a	Confirmed	
☐	☒	The exact sample size (*n*) for each experimental group/condition, given as a discrete number and unit of measurement
☐	☒	A statement on whether measurements were taken from distinct samples or whether the same sample was measured repeatedly
☐	☒	The statistical test(s) used AND whether they are one- or two-sided<br>*Only common tests should be described solely by name; describe more complex techniques in the Methods section.*
☐	☒	A description of all covariates tested
☐	☒	A description of any assumptions or corrections, such as tests of normality and adjustment for multiple comparisons
☐	☒	A full description of the statistical parameters including central tendency (e.g. means) or other basic estimates (e.g. regression coefficient) AND variation (e.g. standard deviation) or associated estimates of uncertainty (e.g. confidence intervals)
☒	☐	For null hypothesis testing, the test statistic (e.g. F, t, r) with confidence intervals, effect sizes, degrees of freedom and P value noted<br>*Give P values as exact values whenever suitable.*
☒	☐	For Bayesian analysis, information on the choice of priors and Markov chain Monte Carlo settings
☒	☐	For hierarchical and complex designs, identification of the appropriate level for tests and full reporting of outcomes
☒	☐	Estimates of effect sizes (e.g. Cohen's d, Pearson's r), indicating how they were calculated

Our web collection on statistics for biologists contains articles on many of the points above.

Software and code

Policy information about availability of computer code

Data collection	Most of the data in this manuscript was generated from this research. We did use published RNA-seq from a previous publication (NCBI GEO datasets (GSE125903)) and published proteomic data (PRIDE: PXD002774).
Data analysis	Each of the dataset generated in this manuscript used standard Data pipeline: RNA-seq: tophat2, quasR and edgeR.<br>Proteome: UNIPROT and Benjamini-Hochberg multiple hypothesis correction.<br>Metabolome: MultiQuant v2.0 software

For manuscripts utilizing custom algorithms or software that are central to the research but not yet described in published literature, software must be made available to editors/reviewers. We strongly encourage code deposition in a community repository (e.g. GitHub). See the Nature Research guidelines for submitting code & software for further information.

Data

Policy information about availability of data

All manuscripts must include a data availability statement. This statement should provide the following information, where applicable:

- Accession codes, unique identifiers, or web links for publicly available datasets
- A list of figures that have associated raw data
- A description of any restrictions on data availability

All the processed Proteomic, RNA-seq and Metabolisms data is included within the supplementary tables. The raw data will be upload to the GEO server once paper is accepted for publication.

Field-specific reporting

Please select the one below that is the best fit for your research. If you are not sure, read the appropriate sections before making your selection.

☒ Life sciences ☐ Behavioural & social sciences ☐ Ecological, evolutionary & environmental sciences

For a reference copy of the document with all sections, see nature.com/documents/nr-reporting-summary-flat.pdf

Life sciences study design

All studies must disclose on these points even when the disclosure is negative.

Sample size	Used 2 different shRNAs targeting RB to control for off-targets.
Data exclusions	Data was excluded from all RNA-seq, proteomics and Metabolite datasets if a 0 value was detected in any sample. In addition, RNA, protein and metabolites below the FPKM cut-off were removed.
Replication	Each experiment was conducted in triplicate from 2 independent groups of cells expressing different shRNAs targeting different regions of RB. As the shRNAs are DOX inducible, we also measured changes in DOX- cells to normalize the data.
Randomization	N/A
Blinding	N/A

Reporting for specific materials, systems and methods

We require information from authors about some types of materials, experimental systems and methods used in many studies. Here, indicate whether each material, system or method listed is relevant to your study. If you are not sure if a list item applies to your research, read the appropriate section before selecting a response.

Materials & experimental systems

n/a	Involved in the study	
☐	☒	Antibodies
☐	☒	Eukaryotic cell lines
☒	☐	Palaeontology
☒	☐	Animals and other organisms
☒	☐	Human research participants
☒	☐	Clinical data

Methods

n/a	Involved in the study	
☒	☐	ChIP-seq
☒	☐	Flow cytometry
☒	☐	MRI-based neuroimaging

Antibodies

Antibodies used	pRB , ATP5E , TrpRS, ADPGK, β-Actin, CS, p-pRB, RBL1, RBL2, S6 and S6K.
Validation	tested in samples using shRNAs and other genomic deletions

Eukaryotic cell lines

Policy information about cell lines

Cell line source(s)	Retinal Pigment Epithelial 1 (RPE1) cells, BJ cells
Authentication	Short tandem repeat sequencing
Mycoplasma contamination	confirmed negative
Commonly misidentified lines (See ICLAC register)	RPE1, BJ cells
